# Blood Biomarker‐Based Predictive Indicator for Liver Metastasis in Alpha‐Fetoprotein‐Producing Gastric Cancer and Multi‐Omics Tumor Microenvironment Insights

**DOI:** 10.1002/advs.202503499

**Published:** 2025-05-28

**Authors:** Yongfeng Ding, Yiran Chen, Jing Zhang, Qingrui Wang, Songting Zhu, Junjie Jiang, Chao He, Jincheng Wang, Laizhen Tou, Jingwei Zheng, Bicheng Chen, Sizhe Hu, Xiongfei Yu, Haohao Wang, Yimin Lu, Mei Kong, Yanyan Chen, Haiyong Wang, Haibin Zhang, Hongxia Xu, Fei Teng, Xian Shen, Nong Xu, Jian Ruan, Zhan Zhou, Jun Lu, Lisong Teng

**Affiliations:** ^1^ Department of Medical Oncology The First Affiliated Hospital School of Medicine Zhejiang University Hangzhou 310000 China; ^2^ Department of Surgical Oncology The First Affiliated Hospital School of Medicine Zhejiang University Hangzhou 310000 China; ^3^ State Key Laboratory of Advanced Drug Delivery and Release Systems & Zhejiang Provincial Key Laboratory of Anti‐Cancer Drug Research College of Pharmaceutical Sciences Zhejiang University Hangzhou 310000 China; ^4^ Department of Gastroenterology Affiliated Hangzhou First People's Hospital Westlake University School of Medicine Hangzhou 310000 China; ^5^ Department of Radiology First Affiliated Hospital School of Medicine Zhejiang University Hangzhou 310000 China; ^6^ Department of Gastrointestinal Surgery Lishui Central Hospital the Fifth Hospital Affiliated to Wenzhou Medical University Lishui 323000 China; ^7^ Department of Gastrointestinal Surgery The Second Affiliated Hospital Wenzhou Medical University Wenzhou 325000 China; ^8^ Department of Gastrointestinal Surgery The First Affiliated Hospital Wenzhou Medical University Wenzhou 325000 China; ^9^ Department of General Surgery Jinyun People's Hospital Lishui 323000 China; ^10^ Department of Gastrointestinal Surgery Dongyang Hospital Affiliated to Wenzhou Medical University Dongyang People's Hospital Jinhua 321000 China; ^11^ Department of Pathology The First Affiliated Hospital School of Medicine Zhejiang University Hangzhou 310000 China; ^12^ Innovation Institute for Artificial Intelligence in Medicine and Liangzhu Laboratory School of medicine Zhejiang University Hangzhou 310000 China; ^13^ Department of Cell Biology School of Medicine Zhejiang University Hangzhou 310000 China

**Keywords:** alpha‐fetoprotein‐producing gastric cancer, immunotherapy, liver metastasis, tumor microenvironment

## Abstract

Alpha‐fetoprotein‐producing gastric cancer (AFPGC) is a rare but highly aggressive subtype of gastric cancer. Patients with AFPGC are at high risk of liver metastasis, and the tumor microenvironment (TME) is complex. A multicenter retrospective study is conducted from January 2011 to December 2021 and included 317 AFPGC patients. Using a multivariable logistic regression model, a nomogram for predicting liver metastasis is built. By combining AFP and the neutrophil–lymphocyte ratio (NLR), we developed a novel and easily applicable predictive indicator, termed ANLiM score, for liver metastasis in AFPGC. An integrated multi‐omics analysis, including whole‐exome sequencing and proteomic analysis, is conducted and revealed an immunosuppressive TME in AFPGC with liver metastasis. Single‐cell RNA sequencing and multiplex immunofluorescence identified the potential roles of tumor‐associated neutrophils and tertiary lymphoid structures in shaping the immune microenvironment. These findings are validated in a real‐world cohort receiving anti‐programmed cell death 1 (anti‐PD‐1) therapy, which showed concordant effectiveness. In addition, the ANLiM score is also identified as a promising biomarker for predicting immunotherapy efficacy. Overall, a blood biomarker‐based predictive indicator is developed for liver metastasis and immunotherapy response in AFPGC. The findings on immune microenvironmental alterations for AFPGC with liver metastasis provide new insights for optimizing immunotherapy strategies.

## Graphical abstract

Alpha‐fetoprotein‐producing gastric cancer (AFPGC), a rare and aggressive subtype of gastric cancer, presents a high risk of liver metastasis. This study introduces the ANLiM score, a blood biomarker‐based predictive indicator for assessing liver metastasis and immunotherapy response. Multi‐omics analyses reveal critical immune microenvironmental alterations in AFPGC with liver metastasis, offering new insights for optimizing immunotherapy strategies.

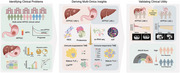

## 1 Introduction

Gastric cancer (GC) is one of the leading causes of cancer‐related death worldwide.^[^ ^1^ ^]^ Alpha‐fetoprotein‐producing gastric cancer (AFPGC) is a rare and distinct subtype of GC that is characterized by the production of AFP and accounts for approximately 1.8% to 7.9% of all GCs.^[^ ^2^, ^3^, ^4^ ^]^ AFPGC is known to be highly aggressive, with a pronounced tendency for lymph node and liver metastasis.^[^ ^5^ ^]^ Previous studies have indicated that liver metastasis is an independent prognostic factor for patients with AFPGC,^[^ ^3^ ^]^ with an incidence of 40–60%,^[^ ^2^, ^3^, ^6^ ^]^ which markedly exceeds the rate of 9.9–18.7% observed in conventional GC.^[^ ^7^, ^8^ ^]^ Currently, the treatment for AFPGC follows the general guidelines for GC, exhibiting a dismal prognosis.^[^ ^4^ ^]^ Given that liver metastasis is a hallmark feature of AFPGC, elucidating its underlying mechanisms is essential and will be pivotal for optimizing future treatment strategies and improving the prognosis.

Cancer has long been considered a genetic disease, with the accumulation of gene mutations being the basis for cancer initiation and metastasis.^[^ ^9^, ^10^ ^]^ However, it is becoming increasingly apparent that genetics alone may not adequately explain the mechanisms of cancer metastasis, as few metastasis‐specific mutations have been definitively identified.^[^ ^11^ ^]^ Thus, cancer geneticists are increasingly focusing on nongenetic variations to seek explanations for metastasis.^[^ ^12^ ^]^ In the past decade, the tumor microenvironment (TME), a key non‐genetic factor, has been reported to play a crucial role in the metastasis of malignant tumors through the spatiotemporal dynamics of its various components (e.g., immune cells and the extracellular matrix).^[^ ^12^, ^13^ ^]^ Thus, identifying the early shifts associated with metastasis within the TME has the potential to revolutionize the early diagnosis and treatment of cancer metastasis, offering new strategies and hope.

Previous studies have suggested that changes in the TME within the primary tumor site encompass early events associated with tumor metastasis.^[^ ^13^ ^]^ For instance, alterations in the local microenvironment of primary tumors, such as extracellular matrix remodeling and altered immune cell composition, establish conditions conducive to tumor dissemination.^[^ ^13^ ^]^ Additionally, during the initial stages of metastasis, the TME within primary tumors can induce systemic immune and inflammatory responses, thereby promoting tumor spread and colonization at distant sites.^[^ ^13^, ^14^ ^]^ Recently, the advent of high‐throughput technologies, including proteogenomics and single‐cell transcriptomics, has propelled cancer research from the single‐genome level to a comprehensive multi‐omics approach, offering new insights into the complexity of the TME.^[^ ^15^, ^16^ ^]^ Therefore, utilizing multi‐omics approaches to characterize the features of TME changes in primary AFPGC lesions may aid in developing new strategies for the early identification and treatment of liver metastasis in AFPGC.

In this study, we aimed to investigate the clinicopathological characteristics of AFPGC and developed a clinical prediction model for liver metastasis. Additionally, we compared the proteogenomic and single‐cell transcriptomics profiles of primary lesions of non‐metastatic AFPGC and those with liver metastasis to elucidate alterations in the TME. Our findings were validated in a real‐world clinical cohort. The insights gained from this study may provide a new theoretical foundation for the early detection and therapeutic intervention of liver metastasis in AFPGC.

## 2 Result

### 2.1 Liver Metastasis was Identified as a Key Clinical Feature of AFPGC, with ANLiM Score Serving as a Predictive Indicator

**Figure 1:**
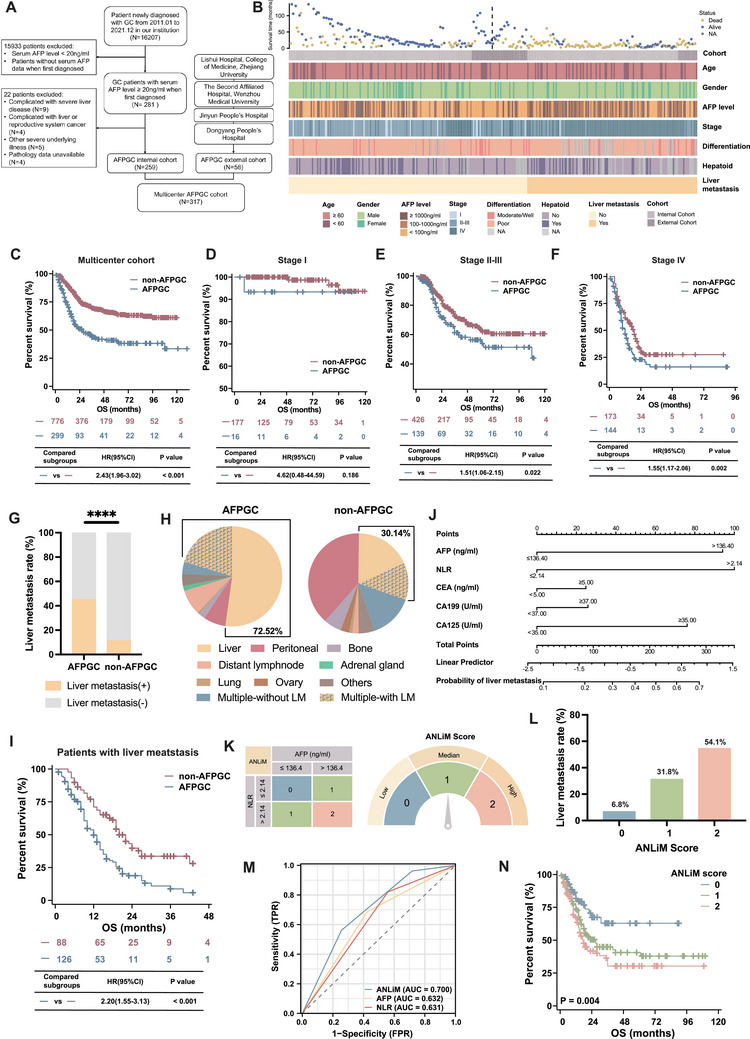
Clinicopathological characteristics of AFPGC cohort and the construction of ANLiM as a predictive biomarker for liver metastasis. A) Screening workflow for the multicenter AFPGC Cohort. B) Distribution of clinicopathological characteristics AFPGC cohort. The survival time and survival status of each patient are displayed above the figure. Comparison of overall survival between AFPGC cohort and non‐AFPGC cohort C), as well as across various TNM stages D–F). G) The incidence of liver metastasis in AFPGC and non‐AFPGC cohorts. H) Comparison of metastatic pattern between two cohorts. I) Comparison of overall survival between two cohorts. J) A nomogram for predicting synchronous liver metastasis of AFPGC. K) Development and calculation of the ANLiM Score. (e.g., serum AFP = 320 ng/mL, NLR = 4.2, yielding ANLiM = 2; serum AFP = 98 ng/mL, NLR = 2.8, yielding ANLiM = 1) L) Relationship between the ANLiM score and the incidence of synchronous liver metastasis. M) Comparison of the predictive ability of ANLiM score with AFP and NLR for Liver Metastasis in AFPGC. N) Survival curves of AFPGC stratified by different ANLiM scores. NA, not available. ^****^ *p* < 0.0001.

To better elucidate the clinicopathological characteristics of AFPGC, we included patients with AFPGC from five hospitals in China to form a multicenter cohort (N = 317, **Figure** **1**A; Table S2, Supporting Information). Figure 1B illustrates the distribution of various clinicopathological characteristics in the multicenter cohort (Table S3, Supporting Information). Additionally, we matched a non‐AFPGC cohort (serum AFP levels at diagnosis <20 ng mL^−1^) at a ratio of ≈1:2.5, based on the admission time. The comparison revealed that AFPGC was characterized by a later stage at diagnosis, a higher proportion of male patients, and poorer differentiation (Table S3, Supporting Information). The overall survival (OS) of the AFPGC cohort was significantly worse than that of the non‐AFPGC cohort (Hazard Ratio [HR] = 2.43, 95% Confidence Interval [CI] = [1.96, 3.02], *p* < 0.001, Figure 1C). Figure 1D–F illustrated that within each stage‐specific subgroup, the prognosis of AFPGC was also relatively poorer than that of non‐AFPGC.

Additionally, patients with AFPGC exhibited a significantly higher incidence of liver metastasis than did those without AFPGC (41.6% vs 11.3%, *p* < 0.001, Figure 1G). Among the initial metastases, liver metastasis constituted 72.5% of cases in AFPGC, which was markedly higher than the 30.1% observed in non‐AFPGC cases (Figure 1H). Moreover, among patients diagnosed with liver metastasis, those with AFPGC exhibited worse OS than of those with non‐AFPGC (HR = 2.20, 95% CI = [1.55, 3.13], *p* < 0.001, Figure 1I). These results indicated that liver metastasis is a prominent clinical feature of AFPGC. Early identification of liver metastasis and prompt intervention may significantly improve the prognosis of patients with AFPGC. Currently, no clinical prediction model exists for liver metastasis in AFPGC. Based on the univariate analysis (Table S4, Supporting Information), we developed a nomogram based on clinically feasible variables to predict synchronous liver metastasis (liver metastasis occurring at the time of diagnosis or within six months thereafter) (Figure 1J; Figure S1, Supporting Information). To facilitate clinical application, we extracted the two most significant variables from the predictive model, AFP and NLR (neutrophil–lymphocyte ratio), to create a novel index termed the ANLiM (AFP and NLR in Liver Metastasis of AFPGC) (Figure 1K). A score of 0 is given if AFP is less than or equal to 136.4 ng mL^−1^ and NLR is less than or equal to 2.14. If AFP is less than or equal to 136.4 ng mL^−1^ but NLR is greater than 2.14, the score is 1. When AFP exceeds 136.4 ng mL^−1^ and NLR is less than or equal to 2.14, the score is also 1. Finally, a score of 2 is assigned if both AFP and NLR are greater than their respective thresholds of 136.4 ng mL^−1^ and 2.14. When the ANLiM score was 0, the likelihood of synchronous liver metastasis was 6.8%, which increased to 31.8% and 54.1% when the ANLiM scores were 1 and 2, respectively (Figure 1L). The predictive performance of the ANLiM score for synchronous liver metastasis was significantly superior to that of AFP or NLR alone (Figure 1M). The predictive value of the ANLiM score for synchronous liver metastasis was further validated in the validation cohort (Table S5; Figure S2, Supporting Information). Additionally, a higher ANLiM score was associated with poorer OS (*p* = 0.004, Figure 1N).

### 2.2 Characterization of Multi‐Omics Features Related to Liver Metastasis in AFPGC and Key Findings on the Immunosuppressive Microenvironment

**Figure 2:**
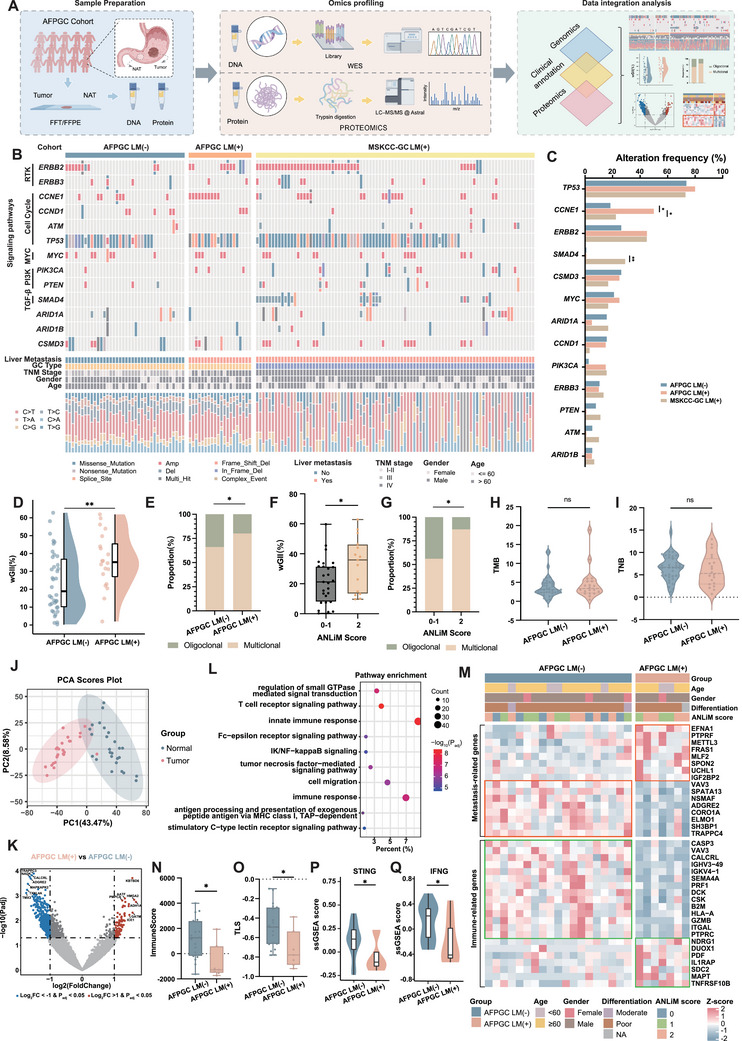
Characterization of molecular features and microenvironmental changes associated with liver metastasis in AFPGC. A) Schematic workflow for genomic sequencing and proteomic analysis. B) The landscape of gene alterations among AFPGC LM (−), AFPGC LM (+), and MSKCC‐GC LM (+) groups. The middle panel displays somatic mutations across genes (rows) and tumor samples (columns), while the left panel annotates cancer‐related signaling pathways for these genes. The bottom panel presents different clinicopathological features. C) Comparison of gene alteration frequencies among different groups. D,E) Comparison of wGII index D) and polyclonality E) between two groups. F,G) Comparison of wGII index (F) and polyclonality G) between high ANLiM score and low ANLiM score groups. H,I) Comparison of TMB H) and TNB I) between two groups. J) PCA analysis of AFPGC tumor samples and adjacent normal tissues. K) Volcano plot of DEPs between two groups. L) Pathway enrichment analyses of DEPs. M) Heatmap of DEPs associated with immune process or metastasis in AFPGC LM(−) and AFPGC LM(+) groups. N,O) Comparison of ImmuneScores N) and TLS scores O) between AFPGC LM(−) and AFPGC LM(+) groups. P,Q) Comparison of ssGSEA enrichment scores for STING P) and IFNG Q) pathways. LM, liver metastasis; NAT, normal adjacent tissue; FFT, fresh frozen tissue; FFPE, formalin‐fixed paraffin‐embedded tissue; LM(−), the absence of liver metastasis; LM(+), the presence of liver metastasis; RTK, Receptor Tyrosine Kinase; wGII, weighted genomic instability index; PCA, principal component analysis; ssGSEA, single‐sample gene set enrichment analysis; NA, not available. ^*^ *p* < 0.05; ^**^ *p* < 0.01; ns, no significance.

Liver metastasis is a critical event in AFPGC progression; however, the underlying molecular mechanisms remain unclear. We leveraged specimens from an AFPGC cohort that had been collected over the past decade for genomic sequencing and proteomic analyses (**Figure** **2**A). Based on the presence or absence of liver metastasis, the AFPGC genomic cohort was divided into a liver metastasis group (AFPGC LM[+], N = 20) and a non‐tumor‐associated neutrophils liver metastasis group (AFPGC LM[−], N = 38). We also included a cohort of common GC with liver metastasis from Memorial Sloan Kettering Cancer Center (MSKCC) (MSKCC‐GC LM[+], N = 89). Figure 2B illustrates the landscape of the somatic mutations and copy number variations in several cancer‐related signaling pathways as well as in driver genes. The alteration frequencies of most cancer‐related genes were not significantly different among the three groups (Figure 2C; Table S6, Supporting Information). However, CCNE1 alteration (primarily gene amplification) was significantly more frequent in the AFPGC LM (+) group. Chromosomal instability (CIN), to some extent, reflects the invasive ability of tumors. We found that the CIN score, measured using weighted genome integrity index (wGII, details in Supporting Methods), was significantly elevated in the AFPGC LM(−) group (Figure 2D). Additionally, the AFPGC LM(−) group exhibited a higher proportion of polyclonality (Figure 2E). Notably, higher ANLiM scores were significantly correlated with higher wGII values (Figure 2F) and a greater proportion of polyclonality (Figure 2G). Conversely, no significant differences were observed in tumor mutation burden (TMB) (Figure 2H) and tumor neoantigen burden (TNB) (Figure 2I) between the two groups.

Currently, no proteomics studies have specifically addressed AFPGC. In this study, we performed proteomics analyses of 52 samples (comprising cancerous and adjacent noncancerous tissues) from 26 patients with AFPGC (Figure S3, Supporting Information). Using PCA, we clearly distinguished between cancerous and adjacent non‐cancerous AFPGC samples (Figure 2J). We compared the differences in protein expression levels between AFPGC LM(+) and LM(−) groups, identifying 518 differentially expressed proteins (DEPs) (Figure 2K; Table S7, Supporting Information). Pathway enrichment analyses revealed that the DEPs were mainly involved in cell migration and immune‐related pathways (Figure 2L). Figure 2M illustrates the differences in protein expression of immune‐related genes and genes associated with liver metastasis in digestive system tumors between the AFPGC LM(−) and AFPGC LM(+) groups (Figure 2M; Supporting Methods). The AFPGC LM(+) group exhibited significantly lower immune scores (Figure 2N) and reduced tertiary lymphoid structure (TLS) scores (Figure 2O), indicating the presence of a pronounced immunosuppressive microenvironment within the primary tumor lesions of the AFPGC LM(+) group. Recent studies have shown that the suppressed stimulator of interferon genes (STING)^[^ ^17^ ^]^ and interferon‐gamma (IFNG) pathways^[^ ^18^ ^]^ play crucial roles in the immunosuppressive microenvironment of tumors. We also observed reduced activity of these pathways in the AFPGC LM(+) group. (Figure 2P,Q).

### 2.3 Single‐Cell Transcriptomics Revealed the Involvement of TANs in Shaping the Immunosuppressive Microenvironment in AFPGC Patients with Liver Metastasis

**Figure 3:**
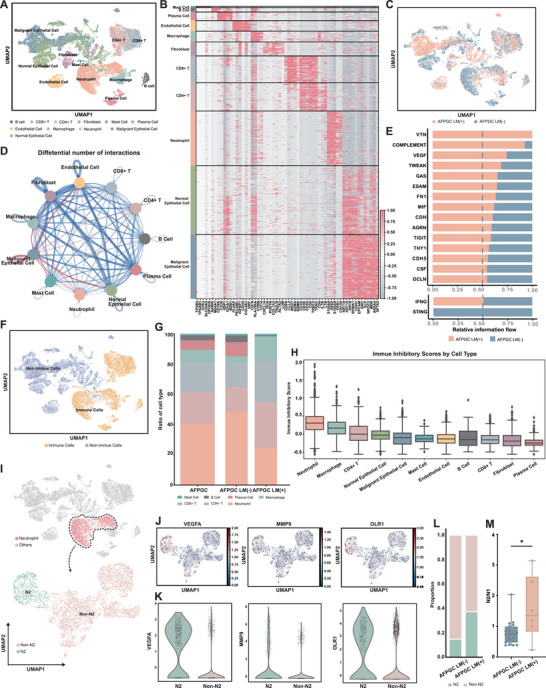
Single‐cell analysis reveals immune cells in the tumor microenvironment of AFPGC. A) UMAP plots depicting distinct cell types present in AFPGC. B) Gene expression heatmap in distinct cell types. C) UMAP plots showing cell clustering, colored with AFPGC LM(−) and AFPGC LM(+). D) The differences in the number of interactions between AFPGC LM(+) group and LM(−) group. Red (blue) edges indicate that the number of interactions increases (decreases) in the LM(+) group. E) Active Receptor‐Ligand Pathways in AFPGC LM(+) and AFPGC LM(−). F) UMAP plots showing cell clustering, colored with immune cells and non‐immune cells. G) Proportional distribution of different immune cells in AFPGC. H) Immune‐inhibitory scores of each cell type in AFPGC. I) Neutrophils were divided into 2 clusters (N2 and non‐N2) using UMAP plot. J,K) Expression of VEGFA, MMP9, and OLR1 in N2 and non‐N2 clusters. L) Differential distribution of N2 and non‐N2 subsets among TANs in AFPGC with and without LM. M) The comparison of N2/N1 ratio between AFPGC LM(−) and AFPGC LM(+) based on ssGSEA analysis using proteomics data. UMAP, uniform manifold approximation and projection; TANs, tumor‐associated neutrophils; ^*^ *p* < 0.05.

To gain a deeper understanding of the changes occurring in the TME of primary AFPGC lesions during liver metastasis, we analyzed single‐cell sequencing samples from four AFPGC cases (Table S8, Supporting Information). The UMAP plot shows the identified cell types, including epithelial cells (malignant/normal epithelial cells), fibroblast cells, CD4+ T cells, CD8+ T cells, macrophage cells, and neutrophil cells (**Figure** **3**A). The expression of representative genes across the different cell types is shown in Figure 3B. We also demonstrated the expression levels of AFP in different cell types and found that it is primarily expressed in malignant epithelial cells (Figure S4, Supporting Information). Figure 3C illustrates the distribution of cells from the AFPGC LM(+) and AFPGC LM(−) groups. The total number and intensity of interactions between cell clusters were identified using CellChat (Figure S5, Supporting Information). Additionally, we showed the differences in the number and intensity of interactions between the AFPGC LM(+) and LM(−) groups (Figure 3D; Figure S6, Supporting Information). All communication probabilities in the information network were summarized, and receptor‐ligand pathways that were more active in the AFPGC LM(+) group were shown (Figure 3E). In addition, we specifically examined the STING and IFNG pathways and confirmed that the STING pathway was notably inactive in the AFPGC LM(+) group (Figure 3E).

The AFPGC LM(+) group exhibited a lower immune score (Figure S7, Supporting Information). Based on the cell clustering results, the cells were classified as immune or non‐immune cells (Figure 3F). Among the immune cells, neutrophils, CD4+ T cells, and CD8+ T cells accounted for 40.5%, 21.1%, and 19.8% of the cells, respectively (Figure 3G; Table S9, Supporting Information). Neutrophils presented the highest immune‐inhibitory scores (details in Supporting Methods) among the tumor‐infiltrating immune cells, followed by macrophages and CD4+ T cells (Figure 3H). We then re‐clustered the neutrophils and divided them into N2 (pro‐tumor neutrophil) and non‐N2 neutrophils (Figure 3I). VEGFA, MMP9, and OLR1, which are classic markers of N2 neutrophils, were mainly expressed in the N2 clusters (Figure 3J,K). Additionally, we mapped the distribution and expression patterns of CD54 (ICAM1) and CD177 as markers associated with N1 neutrophils, as well as IFIT1 and RSAD2 as markers of interferon‐stimulated neutrophils (Figure S8, Supporting Information). Notably, the immune inhibitory scores for the N2 neutrophils that we identified were significantly higher than those for the non‐N2 neutrophils (Figure S9, Supporting Information). This suggests that N2 has an important role in shaping the immunosuppressive environment in AFPGC. The relative proportion of N2, as a subset of tumor‐associated neutrophils (TANs), was higher in AFPGC patients with liver metastasis (Figure 3L). We also performed ssGSEA analysis using the proteomics data and confirmed that the N2/N1 ratio was higher in the AFPGC LM(+) group (Figure 3M).

### 2.4 Dynamics of the Immune Microenvironment of AFPGC Verified by mIF

**Figure 4:**
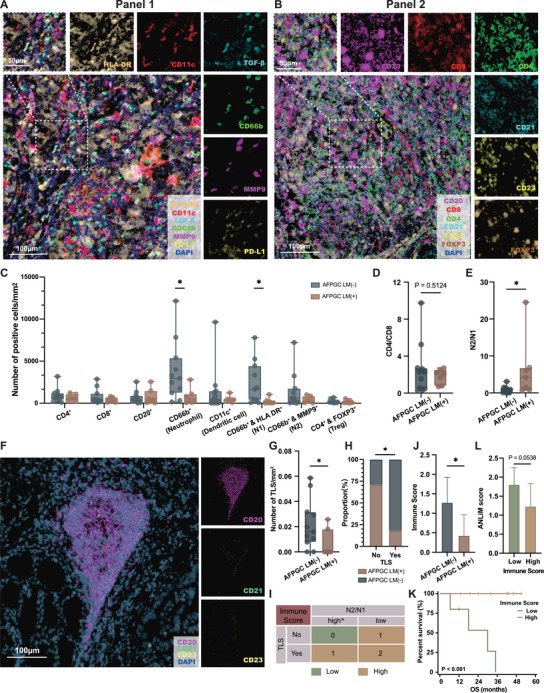
Comparison of tumor immune microenvironment of AFPGC using mIF. A) Representative mIF images in AFPGC stained for antibodies in panel 1. Panel 1 consisted of CD66b, HLA‐DR, CD11c, MMP9, TGF‐β and PD‐L1. B) Representative mIF images in AFPGC stained for antibodies in panel 2. Panel 2 consisted of CD4, CD8, FoxP3, CD20, CD21 and CD23. C) Box plots showing the cell density (number of cells per mm^2^) between AFPGC LM(−) and AFPGC LM(+). D,E) Comparison of CD4/CD8 ratio D) and N2/N1 ratio E) between AFPGC LM(−) and AFPGC LM(+). F) A typical image of a mature tertiary lymphoid structure using mIF. G,H) Comparison of the density G) and TLS positivity H) between AFPGC LM(−) and AFPGC LM(+). I) Calculation of the mIF‐based immune score. * represents N2/N1 > 0.8, which refers to the result of ssGSEA analysis using proteomics data. (e.g., The presence of TLS, N2/N1 ratio = 0.9, yielding score = 1) J) Comparison of mIF‐based immune score between AFPGC LM(−) and AFPGC LM(+) groups. K) Comparison of overall survival between high and low immune score groups. L) The relation between mIF‐based immune score and ANLiM scores. mIF, multiplex immunofluorescence; ^*^ *p* < 0.05.

To further verify the correlation between the tumor immune microenvironment and liver metastasis of AFPGC, we conducted multiplex immunofluorescence (mIF) on 18 clinical samples, including 11 cases without liver metastasis (LM[−]) and seven cases with liver metastasis (LM[+]). We established two mIF panels to profile T‐cell subpopulations, B‐cell subpopulations, TANs subtypes (N1 and N2), dendritic cells, TLSs (**Figure** **4**A,B). Figure 4C compares the differences in cell density between the AFPGC LM(−) and AFPGC LM(+) groups. No significant difference in CD4+/CD8+ was observed between the AFPGC LM(−) and LM(+) groups (Figure 4D); however, the N2/N1 ratio was higher in AFPGC LM(+) than that in LM(−) (Figure 4E). Proteomic analysis revealed a reduced TLS score in AFPGC LM(+), and using mIF, we assessed TLS in a more visual and quantitative manner. Figure 4F shows a typical image of a mature TLS. The TLS density and presence ratio in the AFPGC LM(+) group were both lower than those in the AFPGC LM(−) group (Figure 4G,H). To better characterize the immune microenvironment in AFPGC, we designed a new mIF‐based metric called the “Immune Score”, using the N2/N1 ratio and TLS as indicators (Figure 4I). The liver metastasis group exhibited a lower Immune Score compared to the non‐liver metastasis group (Figure 4J), and patients with lower Immune Scores had significantly poorer OS (Figure 4K). Moreover, we observed that the high Immune Score group tended to have lower ANLiM scores (*p *= 0.054; Figure 4L).

### 2.5 Real‐World Survival Analysis Reveals the Efficacy of Immunotherapy in AFPGC Patients and ANLiM Score can Serve as a Predictive Marker

**Figure 5:**
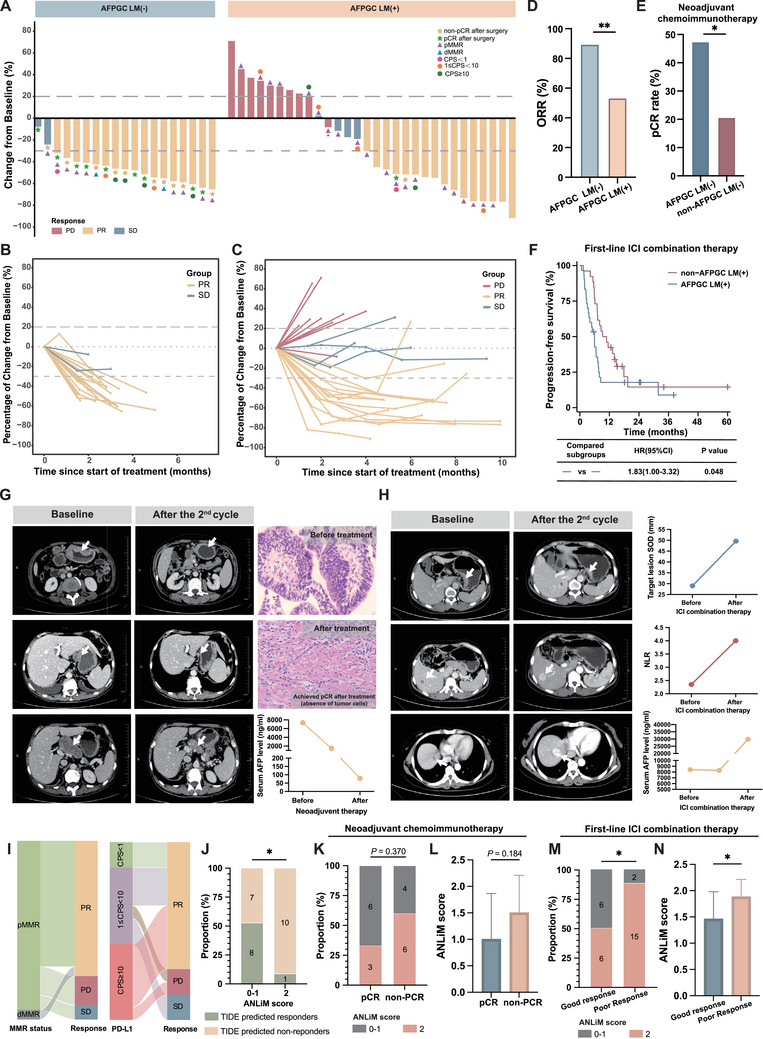
The efficacy of immunotherapy in AFPGC patients and the role of ANLiM as a predictive marker. A) Efficacy of immunotherapy (anti‐PD‐1 therapy) in combination with chemotherapy in AFPGC LM(−) and AFPGC LM(+) groups, respectively. B,C) Spider plots showing treatment responses for all patients in AFPGC LM(−) group. B) and in AFPGC LM(+) group C). D) The ORR for immunotherapy in combination with chemotherapy in AFPGC LM (−) and AFPGC LM (+) patients, respectively. E) Comparison of pCR rate of neoadjuvant chemoimmunotherapy between AFPGC LM(−) and non‐AFPGC LM(−). F) Comparison of PFS between AFPGC LM(+) and non‐AFPGC LM(+) patients receiving first‐line ICI combination therapy. G) Representative case showing reduced tumor lesions of AFPGC LM(−) after immunotherapy‐based neoadjuvant treatment and achieving pCR. ’BASELINE’ indicates the starting point for comparison. H) Representative case showing tumor progression of AFPGC LM(+) after 2 cycles immunotherapy‐based treatment. I) Sankey diagram illustrating the relationships between different MMR statuses, PD‐L1 expression levels, and therapeutic efficacy. J) Association between ANLiM score and TIDE predicted response. K,L) Association between ANLiM score and response to neoadjuvant chemoimmunotherapy in AFPGC LM(−). M,N) Association between ANLiM score and response to first‐line ICI combination therapy in AFPGC LM(−). PD, progressive disease; SD, stable disease; PR, partial response; ORR, objective response rate; pMMR, proficient mismatch repair; dMMR, deficient mismatch repair; CPS, combined positive score; ICI: immune checkpoint inhibitor; SOD, sum of longest diameters; ^*^ *p* < 0.05; ^**^ *p* < 0.01.

To validate the clinical implications identified by multi‐omics analysis, we conducted an efficacy analysis of immunotherapy (anti‐PD‐1 therapy) in real‐world patients with AFPGC (N = 49, Table S10, Supporting Information and Supporting Methods). The waterfall plot in **Figure** **5**A shows the best response to immunotherapy‐based neoadjuvant and first‐line therapies in two groups, respectively. Additionally, Figure 5B,C illustrate the changes in the sum of the diameters of target lesions (tumor burden) over time during treatment for AFPGC LM(−) and AFPGC LM(+), respectively. We observed that the decreased tumor burden in AFPGC patients with liver metastasis was not well maintained over time. In addition, the objective response rate (ORR) for AFPGC LM (−) was higher than that for AFPGC LM (+) (Figure 5D). From another perspective, AFPGC LM (−) patients undergoing combined neoadjuvant immunotherapy exhibited a higher pathological complete response (pCR) rate than that of non‐AFPGC LM (−) patients (Figure 5E). Additionally, AFPGC LM (+) patients demonstrated poorer progression‐free survival (PFS) in first‐line combined immunotherapy compared to non‐AFPGC LM (+) patients (Figure 5F). This finding not only suggests that the efficacy of immunotherapy differs in AFPGC cases compared to non‐AFPGC cases, but also indirectly supports our hypothesis that, based on our understanding of the immune microenvironment in AFPGC, the effectiveness of immunotherapy in AFPGC LM (+) patients is inferior to that in AFPGC LM (−) patients. Figure 5G depicts the case of an AFPGC LM (−) patient who experienced a reduction in tumor size following neoadjuvant immunotherapy and subsequently achieved a pCR after surgery. In contrast, Figure 5H presents the case of an AFPGC LM (+) patient in whom both liver metastases and tumor markers progressed after two cycles of treatment. We evaluated the capacity of the mismatch repair (MMR) status and programmed cell death‐ligand 1 (PD‐L1) expression to predict immunotherapy efficacy and found that the correlation was relatively weak (Figure 5I). Therefore, identification of valuable predictive biomarkers is urgently required. Based on the AFPGC proteomic sequencing cohort in this study, a more in‐depth analysis was conducted. Our findings revealed a significant correlation, showing that a high proportion of patients with elevated ANLiM scores (specifically, ANLiM score = 2) were predicted to be non‐responders according to the Tumor Immune Dysfunction and Exclusion (TIDE) analysis (Figure 5J, the details of TIDE analysis can be found in Supporting Methods). Among patients with AFPGC who were undergoing neoadjuvant immunotherapy, we observed that those who achieved pCR tended to exhibit a lower proportion of high ANLiM scores (Figure 5K) and lower overall ANLiM scores (Figure 5L); however, these findings were not statistically significant. Notably, we observed that with first‐line combined immunotherapy for AFPGC LM(+), the proportion of patients with high ANLiM scores was significantly higher in the poor response group (PFS < 6 months) than in the good response group (PFS ≥ 6 months) (Figure 5M). Additionally, the overall ANLiM score was also higher in the poor response group (Figure 5N). This result suggests that the ANLiM score may have potential predictive value for the immunotherapy efficacy in AFPGC patients, particularly in cases involving liver metastasis.

## 3 Discussion

AFPGC is a unique subtype of GC, and our preliminary research pioneered the reporting of its genomic characteristics of AFPGC.^[^ ^2^ ^]^ In this study, we observed that the incidence of liver metastasis in AFPGC was significantly higher than that in non‐AFPGC. This observation aligns with the results of other studies.^[^ ^3^, ^6^ ^]^ Currently, the diagnosis and treatment of AFPGC follow the guidelines established for conventional GC. Surgery is the primary treatment modality for localized AFPGC. However, a substantial number of patients with AFPGC experience rapid postoperative liver recurrence, posing significant clinical challenges.^[^ ^4^ ^]^ Previous studies have indicated that plasma AFP levels may serve as an effective marker for predicting liver metastasis in AFPGC^[^ ^4^, ^19^ ^]^ In this study, we introduced the ANLiM score for predicting synchronous liver metastasis (including those diagnosed with occult liver metastasis), which demonstrated significantly higher predictive accuracy than that of the plasma AFP levels. Accurate risk prediction of liver metastasis is crucial for making informed clinical decisions and improving prognosis. However, the underlying mechanisms of liver metastasis remain elusive. Therefore, urgent and in‐depth mechanistic investigations are warranted to develop precise therapeutic strategies and enhance the prognosis of AFPGC.

Although certain individual genetic changes have been implicated in the development of metastasis, few appear to be metastasis‐specific.^[^ ^11^, ^20^ ^]^ Previous studies suggest that metastasis may rely more on non‐genetic mechanisms, such as the TME, than previously recognized.^[^ ^12^, ^21^ ^]^ The TME encompasses diverse immune cell types, fibroblasts, the endothelium, and various other tissue‐resident cell types.^[^ ^13^ ^]^ Tumors and their surrounding microenvironment are increasingly being conceptualized as a comprehensive ecosystem and are explored at three different levels: primary (the primary site), regional (metastatic lymph node), and distal (metastatic lesion) onco‐spheres.^[^ ^13^, ^22^, ^23^ ^]^ A comparative analysis of the ecosystems of metastatic and primary tumor sites is necessary for revealing metastasis‐related spatiotemporal heterogeneity.^[^ ^13^, ^24^, ^25^ ^]^ Such insights are crucial for developing novel therapeutic targets and for tackling challenges associated with primary and secondary drug resistance. Tumor metastasis is a complex, multi‐step process that involves multiple stages. During the early stages of tumor metastasis, significant features that promote metastasis often accumulate at the primary site as the primary tumor continues to evolve^[^ ^13^, ^25^ ^]^ Therefore, investigating the alterations in the microenvironment of the primary tumor before and after metastasis may assist with identifying earlier metastasis‐related events at the primary site. Additionally, this will provide valuable insights for the early prediction of tumor metastasis and intervention. In this study, we observed that primary tumors with liver metastasis exhibited higher CIN. CIN arises from persistent errors in chromosomal segregation during mitosis and plays a significant role in cancer metastasis.^[^ ^26^, ^27^ ^]^ The proteome is more closely aligned with biological phenotypes than are either the genome or the transcriptome. Our proteomic analysis revealed that proteins related to metastasis, such as METTL3^[^ ^28^ ^]^ and IGF2BP2^[^ ^29^ ^]^ were highly expressed in the primary lesions of AFPGC with liver metastasis. Interestingly, METTL3 and IGF2BP2 are key members of the m6A methylation machinery, with METTL3 acting as a methylation writer and IGF2BP2 as a reader^[^ ^30^, ^31^ ^]^ Dysregulated m6A methylation has been reported to play a significant role in the remodeling of the TME and promoting tumor metastasis.^[^ ^32^ ^]^ In this study, in addition to the metastasis‐related pathways, we observed that additional immune‐related pathways were enriched through the pathway enrichment analysis of DEPs. Integrated analysis revealed that AFPGC patients with liver metastases exhibited not only significantly lower immunoscores in primary tumors but also higher CIN levels,^[^ ^26^, ^27^ ^]^ both of which converge toward a possible immunosuppressive microenvironment.

To further investigate the immune microenvironment within AFPGC patients, we analyzed the scRNA‐seq data from primary tumors with or without liver metastasis. Our analysis of immune cell components revealed that TANs were represented the predominant cell type within the TME. TANs exhibit multifaceted characteristics and can affect the tumors in different ways depending on their polarization states.^[^ ^33^ ^]^ Analogous to the M1/M2 classification of macrophages, the N1 and N2 have been introduced to define neutrophils with antitumor and pro‐tumor functions, respectively.^[^ ^33^, ^34^ ^]^ The role of macrophages within the immunosuppressive microenvironment has been extensively characterized and well‐documented.^[^ ^35^ ^]^ Our study showed that TANs, especially the N2 subtype (pro‐tumor neutrophil), exhibited higher immune inhibitory scores than macrophages in AFPGC. Recent studies have revealed that TANs, especially the N2 subtype, play a crucial role in establishing an immunosuppressive microenvironment within tumors.^[^ ^36^, ^37^, ^38^ ^]^ The stimulator of interferon genes (STING) pathway plays a crucial role in antiviral host defense mechanisms and antitumor immunity.^[^ ^39^, ^40^ ^]^ Notably, we observed an increased proportion of N2 and suppression of STING signaling in primary tumors with liver metastasis. The biological significance and potential mechanistic role of this finding remain to be further elucidated. Hao et al. utilized neutrophils as carriers to deliver STING agonists for penetrating solid tumors, effectively activating the STING pathway.^[^ ^41^ ^]^ This activation reprograms the TME by converting neutrophils and macrophages into antitumor phenotypes (N1/M1), thereby enhancing antitumor immunity. Lu et al. found that activation of the STING pathway by MnO₂ nanoparticles promotes IRF3 phosphorylation and stimulates IFN‐β secretion in breast cancer xenograft model, thereby inducing a shift in neutrophil polarization from the N2 to the N1 phenotype and enhancing antitumor immunity.^[^ ^42^ ^]^ Additionally, previous studies have indicated that the STING pathway can induce changes in the downstream TGF‐β pathway,^[^ ^43^ ^]^ which has been reported to mediate the N1‐N2 conversion.^[^ ^34^ ^]^ However, the potential association between STING pathway activation and N1/N2 neutrophil polarization dynamics in AFPGC with liver metastases remains to be experimentally explored. For example, future studies could explore the polarization of neutrophils in models with activated STING pathways and assess the resulting alterations of cytokine profiles in AFPGC with liver metastases. Additionally, these studies could investigate the interplay between STING and related signaling pathways, such as TGF‐β, in modulating neutrophil function and phenotype. TLSs are critical indicators of the TME status and serve as essential sites for the initiation of anti‐tumor immune responses.^[^ ^44^, ^45^ ^]^ TLSs modulate the immune microenvironment by actively recruiting circulating immune cells and enhancing local immune responses.^[^ ^44^, ^45^, ^46^ ^]^ Our study showed that the density of TLSs was relatively low in the AFPGC with liver metastasis, indicating limited benefit from immunotherapy. Chelvanambi et al. found that intratumoral injection of the STING agonist can slow melanoma growth, improve vascular normalization, and promote the formation of TLS within the tumor microenvironment, thereby enhancing the anti‐tumor immune response.^[^ ^47^ ^]^ In future studies, the relationship between the formation of TLS and the efficacy of immunotherapy in AFPGC patients with liver metastasis warrants further exploration.

With the widespread use of immune checkpoint inhibitors (ICIs), immune‐based combination therapies have shown significant efficacy in GC, and their application is now being extended from advanced stages to the perioperative setting.^[^ ^48^ ^]^ Our real‐world cohort analysis showed that the efficacy of immune combination therapy was suboptimal in the AFPGC liver metastasis group compared with that in the LM(−) group. This phenomenon can be partially attributed to the characteristics of the immunosuppressive microenvironment. Currently, the most commonly used predictive biomarkers for ICI efficiency are PD‐L1, MMR, TMB, and TNB, all of which are tissue‐based biomarkers.^[^ ^49^ ^]^ Holder et al. previously highlighted the challenges associated with tissue‐based biomarkers, such as invasive procedures, high costs, and heterogeneous sampling. Additionally, our data also indicated that the correlation between PD‐L1, MMR, and the efficacy of immunotherapy for AFPGC was relatively poor. Blood‐based biomarkers might represent promising avenues for future research.^[^ ^49^ ^]^ ANLiM, a novel blood‐based biomarker, was initially proposed as a potential predictor of liver metastasis. Our findings suggest that ANLiM is a promising biomarker for predicting the efficacy of immunotherapy in patients with AFPGC. By integrating AFP, a well‐established tumor marker with confirmed relevance in AFPGC^[^ ^2^, ^19^ ^]^ that reflects immune system dynamics,^[^ ^50^ ^]^ with NLR, an indicator of systemic inflammation,^[^ ^51^ ^]^ ANLiM offers a comprehensive view of the tumor's biological aggressiveness and the host's immune response. Similarly, Scheiner et al. have devised a CRAFITY scoring model that leveraged two clinically established biomarkers: C‐reactive protein and AFP.^[^ ^52^ ^]^ This model offers a rapid and precise means of predicting the therapeutic response to immunotherapy in patients with liver cancer. Therefore, our proposed ANLiM as a robust predictive biomarker for both liver metastasis and the immunotherapy response. This study provides valuable insights for screening patients with AFPGC who may be sensitive to immunotherapy. Additionally, it suggests potential approaches for designing clinical trials and optimizing personalized treatment strategies.

## 4 Limitations

First, this study involved a relatively small number of sequencing samples, encompassing both proteomics and single‐cell sequencing. This is partly attributed to the rarity of AFPGC. However, by focusing on specific clinical issues, we made notable discoveries with the limited available samples. Second, this study did not address interventional research on the immunosuppressive microenvironment in AFPGC patients with liver metastasis, which is a promising direction for future research. In our previous studies, we have constructed multiple humanized patient‐derived xenograft (PDX) models for AFPGC^[^ ^2^ ^]^ and also achieved partial immune reconstruction in PDX mice using peripheral blood mononuclear cells (PBMCs) engraftment from adult donors.^[^ ^53^ ^]^ We are currently developing a patient‐derived xenograft model of AFPGC with a humanized immune system, which will be crucial for future studies. Third, considering that the construction and validation of the ANLiM score are based on retrospective cohorts, it is essential to conduct multi‐center prospective validation in the future. Furthermore, the applicability of the ANLiM score in AFPGC patients accompanied by acute infections requires further investigation.

## 5 Conclusion 

This study established a practical framework for leveraging clinically accessible information and multi‐omics to gain insights into the clinical implications of rare cancers. We developed a blood biomarker‐based predictive indicator for liver metastasis and immunotherapy response in AFPGC. Our findings on immune microenvironmental alterations for AFPGC with liver metastasis provide new insights for optimizing immunotherapy strategies.

## 6 Experimental Section

Details about experimental procedures and data analysis are provided in the Supporting Methods.

## Conflict of Interest

The authors declare no conflict of interest.

## Ethics Approval

This study was approved by the Institutional Review Board of the First Affiliated Hospital of Zhejiang University School of Medicine (approval number: 2024‐0734‐Fast).

## Supporting information



Supporting Information

## Data Availability

The data that support the findings of this study are available on request from the corresponding author. The data are not publicly available due to privacy or ethical restrictions.

## References

[1] F. Bray , M. Laversanne , H. Sung , J. Ferlay , R. L. Siegel , I. Soerjomataram , A. Jemal , CA Cancer J. Clin. 2024, 74, 229. 38572751 10.3322/caac.21834

[2] J. Lu , Y. Ding , Y. Chen , J. Jiang , Y. Chen , Y. Huang , M. Wu , C. Li , M. Kong , W. Zhao , H. Wang , J. Zhang , Z. Li , Y. Lu , X. Yu , K. Jin , D. Zhou , T. Zhou , F. Teng , H. Zhang , Z. Zhou , H. Wang , L. Teng , Nat. Commun. 2021, 12, 3946. 34168152 10.1038/s41467-021-24170-0 PMC8225795

[3] S. Hirajima , S. Komatsu , D. Ichikawa , T. Kubota , K. Okamoto , A. Shiozaki , H. Fujiwara , H. Konishi , H. Ikoma , E. Otsuji , World J. Gastroenterol. 2013, 19, 6055. 24106406 10.3748/wjg.v19.i36.6055 PMC3785627

[4] F. Zhang , Am. J. Cancer Res. 2024, 14, 2124. 38859826 10.62347/IIIO8739 PMC11162674

[5] Y. C. Chang , N. Nagasue , S. Abe , H. Taniura , D. D. Kumar , T. Nakamura , Am. J. Gastroenterol. 1992, 87, 321. 1371637

[6] X. Liu , Y. Cheng , W. Sheng , H. Lu , Y. Xu , Z. Long , H. Zhu , Y. Wang , J. Surg Oncol. 2010, 102, 249. 20740583 10.1002/jso.21624

[7] S. H. Cheon , S. Y. Rha , H.‐C. Jeung , C.‐K. Im , S. H. Kim , H. R. Kim , J. B. Ahn , J. K. Roh , S. H. Noh , H. C. Chung , Ann. Oncol. Off. J. Eur. Soc. Med. Oncol. 2008, 19, 1146. 10.1093/annonc/mdn026 18304963

[8] M. Riihimäki , A. Hemminki , K. Sundquist , J. Sundquist , K. Hemminki , Oncotarget 2016, 7, 52307. 27447571 10.18632/oncotarget.10740 PMC5239553

[9] H. E. Varmus , R. A. Weiss , R. R. Friis , W. Levinson , J. M. Bishop , Proc. Natl. Acad. Sci. U. S. A. 1972, 69, 20. 4333039 10.1073/pnas.69.1.20 PMC427535

[10] L. Angus , M. Smid , S. M. Wilting , J. van Riet , A. Van Hoeck , L. Nguyen , S. Nik‐Zainal , T. G. Steenbruggen , V. C. G. Tjan‐Heijnen , M. Labots , J. M. G. H. van Riel , H. J. Bloemendal , N. Steeghs , M. P. Lolkema , E. E. Voest , H. J. G. van de Werken , A. Jager , E. Cuppen , S. Sleijfer , J. W. M. Martens , Nat. Genet. 2019, 51, 1450. 31570896 10.1038/s41588-019-0507-7 PMC6858873

[11] P. Priestley , J. Baber , M. P. Lolkema , N. Steeghs , E. de Bruijn , C. Shale , K. Duyvesteyn , S. Haidari , A. van Hoeck , W. Onstenk , P. Roepman , M. Voda , H. J. Bloemendal , V. C. G. Tjan‐Heijnen , C. M. L. van Herpen , M. Labots , P. O. Witteveen , E. F. Smit , S. Sleijfer , E. E. Voest , E. Cuppen , Nature 2019, 575, 210. 31645765 10.1038/s41586-019-1689-y PMC6872491

[12] S. Yuan , J. Almagro , E. Fuchs , Nat. Rev. Cancer 2024, 24, 274. 38347101 10.1038/s41568-023-00660-9 PMC11077468

[13] K. E. de Visser , J. A. Joyce , Cancer Cell 2023, 41, 374. 36917948 10.1016/j.ccell.2023.02.016

[14] A. Hoshino , B. Costa‐Silva , T.‐L. Shen , G. Rodrigues , A. Hashimoto , M. Tesic Mark , H. Molina , S. Kohsaka , A. Di Giannatale , S. Ceder , S. Singh , C. Williams , N. Soplop , K. Uryu , L. Pharmer , T. King , L. Bojmar , A. E. Davies , Y. Ararso , T. Zhang , H. Zhang , J. Hernandez , J. M. Weiss , V. D. Dumont‐Cole , K. Kramer , L. H. Wexler , A. Narendran , G. K. Schwartz , J. H. Healey , P. Sandstrom , et al., Nature 2015, 527, 329. 26524530 10.1038/nature15756 PMC4788391

[15] L. Dong , D. Lu , R. Chen , Y. Lin , H. Zhu , Z. Zhang , S. Cai , P. Cui , G. Song , D. Rao , X. Yi , Y. Wu , N. Song , F. Liu , Y. Zou , S. Zhang , X. Zhang , X. Wang , S. Qiu , J. Zhou , S. Wang , X.u Zhang , Y. Shi , D. Figeys , L.i Ding , P. Wang , B. Zhang , H. Rodriguez , Q. Gao , D. Gao , et al., Cancer Cell 2022, 40, 70. 34971568 10.1016/j.ccell.2021.12.006

[16] J. Chen , K. Liu , Y. Luo , M. Kang , J. Wang , G. Chen , J. Qi , W. Wu , B. Wang , Y. Han , L. Shi , K. Wang , X. Han , X. Ma , W. Liu , Y. Ding , L. Wang , H. Liang , L. Wang , J. Chen , Gastroenterology 2023, 165, 88. 36921674 10.1053/j.gastro.2023.03.008

[17] K. R. B. Lanng , E. L. Lauridsen , M. R. Jakobsen , Nat. Immunol. 2024, 25, 1144. 38918609 10.1038/s41590-024-01872-3

[18] A. M. Gocher , C. J. Workman , D. A. A. Vignali , Nat. Rev. Immunol. 2022, 22, 158. 34155388 10.1038/s41577-021-00566-3 PMC8688586

[19] Y.‐K. Wang , L. Shen , X. Jiao , X.‐T. Zhang , World J. Gastroenterol. 2018, 24, 266. 29375212 10.3748/wjg.v24.i2.266 PMC5768945

[20] J. G. Reiter , A. P. Makohon‐Moore , J. M. Gerold , A. Heyde , M. A. Attiyeh , Z A. Kohutek , C. J. Tokheim , A. Brown , R. M. DeBlasio , J. Niyazov , A. Zuker , R. Karchin , K. W. Kinzler , C. A. lacobuzio‐Donahue , B. Vogelstein , M. A. Nowak , Science 2018, 361, 1033. 30190408 10.1126/science.aat7171 PMC6329287

[21] S. Vanharanta , J. Massagué , Cancer Cell 2013, 24, 410. 24135279 10.1016/j.ccr.2013.09.007 PMC3998120

[22] S. J. Horning , Science 2017, 355, 1103. 28302798 10.1126/science.aan1295

[23] X. Chen , E. Song , Cancer Commun. Lond. Engl. 2022, 42, 587. 10.1002/cac2.12316 PMC9257988 35642770

[24] S. Yang , L. Qian , Z. Li , Y. Li , J. Bai , B. Zheng , K. Chen , X. Qiu , G. Cai , S. Wang , H. Huang , J. Wu , Y. Zhu , Q. Zhangyang , L. Feng , T. Wu , R. Wu , A. Yang , K. Wang , R. Wang , Y. Zhang , Y. Zhao , W. Wang , J. Bao , S. Shen , J. Hu , X. Wu , T. Zhou , Z. Meng , W. Liu , et al., Gastroenterology 2023, 164, 407. 36574521 10.1053/j.gastro.2022.11.029

[25] S. Gerstberger , Q. Jiang , G. K. Metastasis , Cell 2023, 186, 1564. 37059065 10.1016/j.cell.2023.03.003 PMC10511214

[26] S. F. Bakhoum , B. Ngo , A. M. Laughney , J.‐A. Cavallo , C. J. Murphy , P. Ly , P. Shah , R. K. Sriram , T. B. K. Watkins , N. K. Taunk , M. Duran , C. Pauli , C. Shaw , K. Chadalavada , V. K. Rajasekhar , G. Genovese , S. Venkatesan , N. J. Birkbak , N. McGranahan , M. Lundquist , Q. LaPlant , J. H. Healey , O. Elemento , C. H. Chung , N. Y. Lee , M. Imielenski , G. Nanjangud , D. Pe'er , D. W. Cleveland , S. N. Powell , et al., Nature 2018, 553, 467. 29342134 10.1038/nature25432 PMC5785464

[27] J. Li , M. J. Hubisz , E. M. Earlie , M. A. Duran , C. Hong , A. A. Varela , E. Lettera , M. Deyell , B. Tavora , J. J. Havel , S. M. Phyu , A. D. Amin , K. Budre , E. Kamiya , J.‐A. Cavallo , C. Garris , S. Powell , J. S. Reis‐Filho , H. Wen , S. Bettigole , A. J. Khan , B. Izar , E. E. Parkes , A. M. Laughney , S. F. Bakhoum , Nature 2023, 620, 1080. 37612508 10.1038/s41586-023-06464-z PMC10468402

[28] Q. Wang , C. Chen , Q. Ding , Y. Zhao , Z. Wang , J. Chen , Z. Jiang , Y. Zhang , G. Xu , J. Zhang , J. Zhou , B. Sun , X. Zou , S. Wang , Gut 2020, 69, 1193. 31582403 10.1136/gutjnl-2019-319639

[29] D. Yu , M. Pan , Y. Li , T. Lu , Z. Wang , C. Liu , G. Hu , J Exp. Clin. Cancer Res. CR 2022, 41, 6. 34980207 10.1186/s13046-021-02212-1 PMC8722037

[30] I. A. Roundtree , M. E. Evans , T. Pan , C. He , Cell 2017, 169, 1187. 28622506 10.1016/j.cell.2017.05.045 PMC5657247

[31] Y. Cheng , Z. Gao , T. Zhang , Y. Wang , X. Xie , G. Han , Y. Li , R. Yin , Y. Chen , P. Wang , J. Hu , T. Zhang , C. Guo , J. Chai , J. Wang , M. Cui , K. Gao , W. Liu , S. Yao , P. Lu , Z. Cao , Y. Zheng , J. Chang , Z. Liu , Q. Song , W. Li , F. Zhou , H. Zhang , Cell Stem Cell 2023, 30, 69. 36574771 10.1016/j.stem.2022.12.003

[32] X. Deng , Y. Qing , D. Horne , H. Huang , J. Chen , Nat. Rev. Clin. Oncol. 2023, 20, 507. 37221357 10.1038/s41571-023-00774-x PMC12466201

[33] T. Keeley , D. L. Costanzo‐Garvey , L. M. Cook , Trends Cancer 2019, 5, 789. 31813456 10.1016/j.trecan.2019.10.013

[34] Z. G. Fridlender , J. Sun , S. Kim , V. Kapoor , G. Cheng , L. Ling , G. S. Worthen , S. M. Albelda , Cancer Cell 2009, 16, 183. 19732719 10.1016/j.ccr.2009.06.017 PMC2754404

[35] J. Li , J. Sun , Z. Zeng , Z. Liu , M. Ma , Z. Zheng , Y. He , W. Kang , Clin. Transl. Med. 2023, 13, 1386. 10.1002/ctm2.1386 PMC10444973 37608500

[36] M. E. Shaul , Z. G. Fridlender , J. Leukoc. Biol. 2017, 102, 343. 28264904 10.1189/jlb.5MR1216-508R

[37] M. A. Giese , L. E. Hind , A. Huttenlocher , Blood 2019, 133, 2159. 30898857 10.1182/blood-2018-11-844548 PMC6524564

[38] R. R. Maas , K. Soukup , N. Fournier , M. Massara , S. Galland , M. Kornete , V. Wischnewski , J. Lourenco , D. Croci , Á. F. Álvarez‐Prado , D. N. Marie , J. Lilja , R. Marcone , G. F. Calvo , R. Santalla Mendez , P. Aubel , L. Bejarano , P. Wirapati , I. Ballesteros , A. Hidalgo , A. F. Hottinger , J.‐P. Brouland , R. T. Daniel , M. E. Hegi , J. A. Joyce , Cell 2023, 186, 4546. 37769657 10.1016/j.cell.2023.08.043

[39] H. Ishikawa , G. N. Barber , Nature 2008, 455, 674. 18724357 10.1038/nature07317 PMC2804933

[40] M. M. M. Tabar , M. Fathi , F. Kazemi , G. Bazregari , A. Ghasemian , Mol. Biol. Rep. 2024, 51, 487. 38578532 10.1007/s11033-024-09418-4

[41] M. Hao , L. Zhu , S. Hou , S. Chen , X. Li , K. Li , N. Zhu , S. Chen , L. Xue , C. Ju , C. Zhang , ACS Nano 2023, 1663, 11764. 10.1021/acsnano.2c11764 36595464

[42] S. Lu , Z. Mi , P. Liu , J. Ding , Y. Ma , J. Yang , P. Rong , W. Zhou , J. Nanobiotechnol. 2024, 22, 443. 10.1186/s12951-024-02726-8 PMC11282601 39068474

[43] C. Chen , P. Xu , Trends Cell Biol. 2023, 33, 630. 36437149 10.1016/j.tcb.2022.11.001

[44] R. Cabrita , M. Lauss , A. Sanna , M. Donia , M. Skaarup Larsen , S. Mitra , I. Johansson , B. Phung , K. Harbst , J. Vallon‐Christersson , A. van Schoiack , K. Lövgren , S. Warren , K. Jirström , H. Olsson , K. Pietras , C. Ingvar , K. Isaksson , D. Schadendorf , H. Schmidt , L. Bastholt , A. Carneiro , J. A. Wargo , I. M. Svane , G. Jönsson , Nature 2020, 577, 561. 31942071 10.1038/s41586-019-1914-8

[45] T. N. Schumacher , D. S. Thommen , Science 2022, 375, abf9419. 10.1126/science.abf9419 34990248

[46] B. A. Helmink , S. M. Reddy , J. Gao , S. Zhang , R. Basar , R. Thakur , K. Yizhak , M. Sade‐Feldman , J. Blando , G. Han , V. Gopalakrishnan , Y. Xi , H. Zhao , R. N. Amaria , H. A. Tawbi , A. P. Cogdill , W. Liu , V. S. LeBleu , F. G. Kugeratski , S. Patel , M. A. Davies , P. Hwu , J. E. Lee , J. E. Gershenwald , A. Lucci , R. Arora , S. Woodman , E. Z. Keung , P.‐O. Gaudreau , A. Reuben , et al., Nature 2020, 577, 549. 31942075 10.1038/s41586-019-1922-8 PMC8762581

[47] M. Chelvanambi , R. J. Fecek , J. L. Taylor , W. J. Storkus , J. Immunother. Cancer 2021, 9, 001906. 10.1136/jitc-2020-001906 PMC7852948 33526609

[48] Y. Y. Janjigian , K. Shitara , M. Moehler , M. Garrido , P. Salman , L. Shen , L. Wyrwicz , K. Yamaguchi , T. Skoczylas , A. Campos Bragagnoli , T. Liu , M. Schenker , P. Yanez , M. Tehfe , R. Kowalyszyn , M. V. Karamouzis , R. Bruges , T. Zander , R. Pazo‐Cid , E. Hitre , K. Feeney , J. M. Cleary , V. Poulart , D. Cullen , M. Lei , H. Xiao , K. Kondo , M. Li , J. A. Ajani , Lancet Lond. Engl. 2021, 398, 27. 10.1016/S0140-6736(21)00797-2 PMC8436782 34102137

[49] A. M. Holder , A. Dedeilia , K. Sierra‐Davidson , S. Cohen , D. Liu , A. Parikh , G. M. Boland , Nat. Rev. Cancer 2024, 24, 498. 38867074 10.1038/s41568-024-00705-7

[50] P. V. Munson , J. Adamik , L. H. Butterfield , Trends Immunol. 2022, 43, 438. 35550875 10.1016/j.it.2022.04.001

[51] A. J. Templeton , M. G. McNamara , B. Seruga , F. E. Vera‐Badillo , P. Aneja , A. Ocaña , R. Leibowitz‐Amit , G. Sonpavde , J. J. Knox , B. Tran , I. F. Tannock , E. Amir , J Natl. Cancer Inst. 2014, 106, dju124. 24875653 10.1093/jnci/dju124

[52] B. Scheiner , K. Pomej , M. M. Kirstein , F. Hucke , F. Finkelmeier , O. Waidmann , V. Himmelsbach , K. Schulze , J. von Felden , T. W. Fründt , M. Stadler , H. Heinzl , K. Shmanko , S. Spahn , P. Radu , A. R. Siebenhüner , J. C. Mertens , N. N. Rahbari , F. Kütting , D.‐T. Waldschmidt , M. P. Ebert , A. Teufel , S. De Dosso , D. J. Pinato , T. Pressiani , T. Meischl , L. Balcar , C. Müller , M. Mandorfer , T. Reiberger , et al., J. Hepatol. 2022, 76, 353. 34648895 10.1016/j.jhep.2021.09.035

[53] W. Lin , Y. Zhang , Y. Yang , B. Lin , M. Zhu , J. Xu , Y. Chen , W. Wu , B. Chen , X. Chen , J. Liu , H. Wang , F. Teng , X. Yu , H. Wang , J. Lu , Q. Zhou , L. Teng , Adv. Sci. 2023, 10, 2303908.

