# Supplementary material for: Blood Biomarker‐Based Predictive Indicator for Liver Metastasis in Alpha‐Fetoprotein‐Producing Gastric Cancer and Multi‐Omics Tumor Microenvironment Insights
Source: Adv Sci (Weinh). 2025 May 28;12(28):e03499. doi: 10.1002/advs.202503499 (PMC12302549; doi:10.1002/advs.202503499)
Supplement: Supplementary file 1 — Supporting Information [file ADVS-12-e03499-s001.pdf]

ADVANCED SCIENCE
Open Access

Supporting Information

for *Adv. Sci.*, DOI 10.1002/advs.202503499

Blood Biomarker-Based Predictive Indicator for Liver Metastasis in Alpha-Fetoprotein-Producing Gastric Cancer and Multi-Omics Tumor Microenvironment Insights

Yongfeng Ding, Yiran Chen, Jing Zhang, Qingrui Wang, Songting Zhu, Junjie Jiang, Chao He, Jincheng Wang, Laizhen Tou, Jingwei Zheng, Bicheng Chen, Sizhe Hu, Xiongfei Yu, Haohao Wang, Yimin Lu, Mei Kong, Yanyan Chen, Haiyong Wang, Haibin Zhang, Hongxia Xu, Fei Teng, Xian Shen, Nong Xu, Jian Ruan, Zhan Zhou*, Jun Lu* and Lisong Teng**

Supplementary methods

Study population and data collection

In this study, we retrieved the electronic medical records of all patients with GC who were admitted to five hospitals (The First Affiliated Hospital of Zhejiang University School of Medicine [ZJU-1], Lishui Hospital, The Second Affiliated Hospital, Jinyun People's Hospital, and Dongyang People's Hospital) between January 2011 and December 2021. The inclusion criteria were 1) confirmation of GC by gastroscopy or postoperative pathological results; 2) serum AFP level > 20ng/mL (exceeding the upper limit of the normal value) before treatment; and 3) complete clinicopathological data. The exclusion criteria were 1) coexistence of other malignant tumors; 2) history of liver diseases, such as acute or chronic hepatitis and liver cirrhosis; and 3) presence of other severe comorbidities that affect survival, such as severe cardiopulmonary diseases and kidney failure. A multicenter cohort of patients with AFPGC who met the inclusion and exclusion criteria was established for this study (multicenter cohort 1). Additionally, a cohort of patients with GC with AFP levels < 20 ng/mL (designated as non-AFPGC) was selected and matched to patients with AFPGC at a ratio of 2.5:1 according to the admission time. Further, using the same inclusion and exclusion criteria, we retrieved the data of patients admitted to ZJU-1 from January 2022 to August 2023 through the hospital's electronic medical record system. Patients with concurrent acute infections were also excluded, defined as those presenting clinical manifestations of specific site infections, positive blood and sputum cultures, and records of antibiotic use. A total of 70 AFPGC cases were included as a validation cohort (independent validation cohort 2).

In order to validate our findings, we included patients from both cohorts (multicenter cohort 1 and independent validation cohort 2) who received combined immunotherapy and chemotherapy between 2019 and 2023. In the multicenter cohort and validation cohort, AFPGC patients who met the following criteria were included in the real-world immunotherapy analysis cohort: 1) Patients received immunotherapy; 2) Patients had early- to mid-stage disease without liver metastasis or late-stage disease with liver metastasis; 3) the baseline tumors of the patients included for assessing immunotherapy efficacy must meet the definitions of measurable target lesions as outlined in RECIST 1.1, specifically: At least one lesion with a longest diameter ≥10 mm on baseline CT/MRI (metastatic lymph nodes required a short-axis diameter ≥15 mm). The final number of patients included was 49.

We retrospectively collected information pertaining to the clinicopathological variables, such as age; sex; serum AFP, CEA, CA125 and CA19-9 levels before treatment; surgical intervention; histological type; tumor location; depth of tumor invasion; lymph node metastasis; mismatch repair (MMR) status; and the programmed cell death-ligand 1 (PD-L1) combined positive score (CPS) from the medical records. The PD-L1 antibody 22C3 (Dako) was selected for detection in this study. The criteria for defining positive PD-L1 expression are as follows: 22C3 positivity is determined by CPS. The CPS is calculated as the ratio of the number of PD-L1-stained cells (including tumor cells, lymphocytes, and macrophages) to the total number of viable tumor cells, multiplied by 100. The neutrophil–lymphocyte ratio (NLR) was calculated as the neutrophil count divided by the lymphocyte count. Tumors were staged according to the eighth edition of the AJCC/UICC TNM staging system. Follow-up data were obtained via phone calls, letters, and the outpatient clinical databases. Overall survival (OS) time was

calculated from the date of diagnosis to the last day of follow-up or the date of death. Progression-free survival (PFS) was calculated from the start of therapy until progression or death. During the follow-up period, we documented the location and timing of the initial metastasis. Synchronous liver metastasis was defined as liver metastasis occurring at the time of diagnosis or within 6 months thereafter, whereas metachronous liver metastasis was defined as liver metastasis that developed more than 6 months after the initial diagnosis.

We retrospectively collected fresh-frozen tumor tissues and paired tumor-adjacent normal tissues from 26 AFPGC patients for protein extraction and proteomics data analysis. Two tumor tissue samples were obtained from patients newly diagnosed with AFPGC with liver metastasis at the ZJU-1 and single-cell RNA sequencing (scRNA-seq) was performed. The scRNA-seq data of the two non-metastasis AFPGC cases were obtained from a study conducted by Mei et al[1]. This study was approved by the Institutional Review Board of the First Affiliated Hospital of Zhejiang University School of Medicine (approval number: 2024-0734-Fast). The preservation and storage of all the data was conducted according to Institutional Review Board.

Genomic data analysis

Somatic mutation and copy number analysis

The whole exome sequencing (WES) data used in this study were obtained from our previously published work[2]. WES libraries were constructed and sequenced on an Illumina platform (150 bp paired-end reads). After removing low-quality reads, adapters, and poly-N sequences, clean reads were aligned to GRCh37 using BWA (v0.7.8). Somatic single nucleotide variants (SNVs) were identified using muTect[3], and somatic insertions and deletions (indels) were detected using Strelka[4]. Somatic copy number alterations (SCNAs) in our datasets were identified using the CNVkit (v0.9.8)[5]. Additionally, genomic alteration data, with annotated clinicopathological information from 89 patients with conventional GC with liver metastasis, were obtained from the Memorial Sloan Kettering Cancer Center (MSKCC) cohort [6] through the cBioPortal database (https://www.cbioportal.org/). An oncoplot displaying the landscape of the somatic mutations and SCNAs was visualized using the R package maftools (v2.10.5)[7].

Calculation of the tumor mutation burden and tumor neoantigen burde

The tumor mutation burden (TMB) was determined by dividing the total number of somatic nonsynonymous mutations divided by the size of the total exome region. For the tumor neoantigen burden (TNB), human leukocyte antigen (HLA) typing was conducted using HLAscan (v2.1.4)[8], and VCF files were generated using the variant effect predictor (VEP) (v104.3)[9]. NetMHCpan (v4.1)[10], MHCflurry (v2.0.0)[11], and DeepHLApan (v.1.1.1)[12], were used to predict neoantigens. The thresholds for each tool were as follows: for NetMHCpan v4.1, identifies binders based on pHLA rankings < 2% or affinity < 500 nM, and we utilized these metrics to filter higher quality neoantigen candidates. MHCflurry provides a rank percentage without a defined threshold, thus we set a 2% cutoff, consistent with NetMHCpan v4.1. For DeepHLApan, which offers posterior probabilities, we defined a threshold at 0.5 for predictions[13]. The intersection of the results from all three predictors was considered the potential neoantigen set. The neoantigen count per mb was then calculated as the TNB[14].

Clonal architecture analysis

To estimate the number of clones in the samples, we analyzed the variant allele frequencies (VAFs), and copy number variation (CNV) data were processed using SciClone (v1.1.0)[15]. Tumors with a single clonal population or a dominant clone with a minor subclone were categorized as the oligoclonal type, whereas tumors with two or more clonal populations were categorized as the multiclonal type. The details of this method have been previously described in our previous publication[16].

Weighted genome integrity index (wGII) analysis

The ploidy of the tumor samples was determined by the weighted median copy number, with weights based on segment lengths. For each sample and 22 autosomes, the gain/loss of genomic material was calculated as a percentage of ploidy. Using percentages eliminates the bias caused by variations in chromosome sizes[17]. The wGII score for a specific sample was calculated as the average percentage value across the 22 autosomes.

Proteomic data acquisition

Protein extraction and quality test

The proteomic samples were prepared and quantified at Novogene (Beijing, China).
Each sample was pulverized in liquid nitrogen and subsequently lysed by adding of SDT solution and DL-Dithiothreitol. Subsequently, the mixture was ultrasonicated for 5 minutes. After incubation at 95°C for 10 minutes, followed by a rapid cooling through an ice bath, the lysate was centrifuged (12,000 g for 15 minutes). The resulting supernatant was then alkylated with an appropriate amount of iodoacetamide in the dark (room temperature). Then, the samples were mixed with acetone by vigorous vortexing and incubated (-20°C). The precipitate was collected (centrifuged at 12,000 g for 15 minutes) and washed with cold acetone. Finally, the pellets were completely dissolved in dissolution buffer (DB buffer). BSA standard protein solution (BSA standards, 0-0.5 g/L) was prepared using protein quantitative kit. BSA standards and sample solution at various dilution multiples were added into a 96-well plate to fill up the volume to 20 μL, with each dilution repeated 3 times. Then, 180 μL of G250 dye solution was quickly added to the plate, which was then placed for 5 minutes. The absorbance at 595 nm was detected. The protein sample was applied to a SDS-PAGE gel for electrophoresis. The gel was stained with coomassie brilliant blue R-250 and subsequently decolorized until the bands became clearly visible.

Protein digestion

The protein samples were individually extracted, and their volume was adjusted to 100 μL using the DB lysis buffer. Subsequently, the trypsin was added to each sample, which was then thoroughly digested (at 37 °C for 4 hour). The mixture was then added with CaCl2 and trypsin, and incubated for approximately 12 hours. The digested mixture was added with formic acid, the pH was adjusted to below 3, and the mixture was subjected to centrifugation at 12,000 g for a period of five minutes. Then the supernatant was gradually introduced to the C18 desalting column, rinsed three times with washing buffer and eluted with elution buffer, and the eluents

from individual sample were collected and lyophilized.

LC-MS/MS analysis-DIA mode

Mobile phase A was prepared as follows: Phase A contained water and 0.1% formic acid. Next, the lyophilized powder was dissolved by 10μL of solution A, and was then subjected to centrifugation (14,000 ×g for 20 minutes, 4°C). Finally, 200 ng of the resulting supernatant was used in the system for liquid quality detection. The Vanquish Neo upgraded UHPLC system was employed, utilizing a C18 pre-column of 174500 and a C18 analytical column of ES906. A Thermo Orbitrap Elite mass spectrometer was employed with an electrospray ionization (ESI) ion source. The acquisition of mass spectra was performed in a data-dependent manner over a full first-stage MS scanning range of m/z 380–980. The primary MS resolution was set at 240,000 (for 200 m/z), with a secondary m/z acquisition range of 150-2000, and the sub-ion resolution for Astral was configured to 80,000. The data obtained from MS assays were integrated into DIA-NN platform to conduct a spectral search to identify both peptides and proteins. Within the DIA-NN, a rigorous filtering process was employed to meticulously sieve the search outcomes, retaining only credible peptide spectrum matches with a confidence threshold of 99% or above, ensuring the highest reliability. Additionally, a rigorous false discovery rate (FDR) was used, rigorously excluding peptides and proteins with an FDR exceeding 1%.

Proteomics data analysis

Pathway enrichment and functional annotation

Principal component analysis (PCA) was used to evaluate the heterogeneity in the protein expression level between tumor and normal samples. To ensure accurate results, the data was standardized by centering the protein expression values by subtracting the mean and scaling them to unit variance. Differentially expressed proteins (DEPs) between the subgroups with or without liver metastasis were identified by the "limma" package in R (adjusted P value < 0.05 and absolute of fold change [FC] > 1.5). Kyoto Encyclopedia of Genes and Genomes (KEGG) enrichment analyses were conducted using the DAVID portal (https://david.ncifcrf.gov/). According to the gene percentage and P value of pathway enrichment, the top ten KEGG pathways were visualized using the R package "ggplot2". The immune-related gene set was derived from ImmPort database[18]. In addition, the liver metastasis associated gene set in digestive system tumors was compiled based on literature mining[19 20 21].

The ESTIMATE algorithm was applied to infer the immune score of each sample[22]. Furthermore, several key immune signatures including the tertiary lymphatic structure (TLS), cyclic GMP-AMP synthase (cGAS) - stimulator of interferon genes (STING), and interferon-gamma (IFN-γ) were estimated using the single-sample gene set enrichment analysis (ssGSEA) algorithm[23].

The combined and deduplicated gene set that resulted from merging the two TLS-related gene sets (the 39-gene set[24 25]and the 9-gene set[26]) collected from previous studies, was used as our TLS-related gene set. The cGAS-STING-related gene set was obtained from the MSigDB[27] (https://www.gsea-msigdb.org). In addition, the IFN-γ-related gene set was extracted from the literature[28].

Tumor Immune Dysfunction and Exclusion (TIDE) analysis

Tumor Immune Dysfunction and Exclusion (TIDE) is a scoring system designed to estimate tumor immune dysfunction and immune exclusion, which encompasses the dysfunction of tumor-infiltrating cytotoxic T-lymphocytes and suppression by immune checkpoints (https://tide.dfci.harvard.edu/)[29]. The TIDE algorithm was employed to process proteomic data and the corresponding clinical information (ANLiM score) from the AFPGC cohort in order to predict potential responses to immunotherapy.

scRNA-seq analysis

Preparation of single-cell suspension and scRNA-seq library

Fresh tissue samples were collected and immediately placed in a special tissue storage medium (Miltenyi Biotech, Germany) before being stored at 4°C. Within 12 hours of collection, the tissues were subjected to a series of procedures to obtain single-cell suspensions. Tissues were then cut into pieces of approximately 1 mm^3 and subjected to enzymatic digestion with incubation (37 °c, 30 minutes). After digestion, the cell suspension was applied to filter to remove any remaining larger particles and then centrifuged (at 300 g for 5 minutes). The cell pellet was carefully re-suspended in DMEM medium. The suspensions with > 80% viability were used for the scRNA-seq assay. Single cell suspensions were loaded into chromium microfluidic chips following the manufacturer's protocol with a 10X Genomics Chromium Single-Cell 3′ Kit (V3). Complementary DNA (cDNA) amplification and sequential library construction were performed according to the manufacturer's instructions. Sequencing was performed with Illumina NovaSeq 6000 sequencing system (PE150).

Quality control and data pre-processing

Cell Ranger Software (v7.1.0) was utilized to initially process the raw scRNA-seq data by demultiplexing, barcode processing, read alignment, quantification of gene expression at the single-cell 3' end, and generation of the expression matrix representing features (genes) linked to the barcodes. The data that passed the initial filtering by Cell Ranger were subsequently employed for further analysis. Further, the Scanpy library (v.1.9.3)[30] was used to process the scRNA-seq data. Cell quality was evaluated by examining the total count of unique molecular identifiers (UMIs) per cell, the total number of detected genes per cell, and the proportion of mitochondrial genes per cell. Low-quality cells were excluded according to the following criteria: 1) Cells identified as outliers in terms of log-transformed total UMI counts, log-transformed number of detected genes, or the percentage of counts in the top 20% of expressed genes, where outliers are defined as those deviating by more than 10 median absolute deviations from the median; and 2) cells with more than 20% of their counts coming from mitochondrial genes. Subsequently, genes that were identified in less than three cells were filtered from the subsequent analyses. For each cell, the count of detected genes was normalized to a target sum of 10^5, followed by a log1p transformation to stabilize variance and improve the interpretability of the data. Gene expression values were scaled to center each gene at zero and standardize variance to one for downstream analyses. This resulted in a working dataset comprising 11,373 cells for AFPGC LM(-) and 10,027 cells for AFPGC LM(+).

Dimension reduction, cell clustering and annotation

We selected the top 2,000 most variable genes identified by the pp.highly_variable_genes implementing the seurat_v3 method for further analysis. Principal component analysis (PCA) was carried out on these genes using the pp.pca function, followed by batch effect mitigation via pp.harmony_integrate. We constructed a neighborhood graph with sc.pp.neighbors, and performed dimensionality reduction and embedding using uniform manifold approximation and projection (UMAP)[31] through tl.umap. Cells were clustered using the Leiden algorithm. To identify the marker genes of each cluster, the Wilcoxon rank-sum test was applied through the tl.rank_genes_groups function. Cell types were manually annotated based on the CellMarker 2.0 database[32], and gene expression profiles were visualized using the pl.rank_genes_groups_heatmap.

Recognition of malignant and non-malignant epithelial cells

InferCNV (v1.11.1)[33] was used to infer CNVs in single-cell gene expression profile and to distinguish malignant cells from normal epithelial cells. The analysis was performed with cluster_by_groups=TRUE, denoise=TRUE, HMM=TRUE, cutoff=0.1, k_obs_groups=2, and sd_amplifier=3. Immune cells in the working dataset were used as cells with normal copy numbers. The inferred large-scale CNVs were clustered using unsupervised clustering (K-means) and visualized in a heatmap. A CNV score for each cell was computed as the mean absolute deviation of expression, and its distribution across clusters was illustrated using violin plots.

Cell-cell interaction analysis

Cell-to-cell crosstalk was inferred using the R package CellChat (v1.6.1)[33] and the CellChat human database. The analysis using CellChat was conducted following the guidelines provided in the CellChat software manual. The "triMean" option for the type parameter was specified during the calculation of the communication probability between different clusters. Then, "netVisual_diffInteraction" was employed to compare the differences in the number or strength of interactions between cell populations in AFPGC LM(+) and AFPGC LM(-). Additionally, the overall information flow within each signaling pathway was compared across groups using rankNet.

Calculation of the immune-inhibitory score

A list of immune inhibitory genes (*CD14, IFRD1, HILPDA, CSTB, IL1R2, ATF3, CEBPB, CD274, NFKBIZ, HSP90AA1, S100A11, CSF3R, RETNLG, LRG1, FOXP3, PTGS2, TNFSF14, S100A9, LILRB3, S100A8, IL10, FCER2, NT5E, IGF2, ARG1, LAg3, NT5E, LILRA1, CTLA4* and *TIGIT*) was compiled based on literature mining results. We performed Z-score normalization for each gene and subsequently calculated the immune inhibitory score by averaging the expression levels of each gene set within each cluster at the single-cell level.

Multiplex immunofluorescence (mIF) staining

Sequential 3-5 μm sections from formalin-fixed paraffin-embedded (FFPE) tissues were prepared for mIF staining. We established two mIF panels to profile specific cell populations such as T-cell subpopulations, B-cell subpopulations and tumor-associated neutrophils (TANs) subtypes in samples from AFPGC patients with or without liver metastasis. Panel 1 consisted of CD66b, HLA-DR, CD11c, MMP9, TGF-β, and PD-L1. Panel 2 consisted of CD4, CD8, FoxP3, CD20, CD21, and CD23. The characteristics of mature TLS include the presence of CD20+ B cells that form prominent follicles, with CD21+ follicular dendritic cells (FDCs) located within or adjacent to these follicles, and CD23+ germinal center cells found in specific regions within the follicles[25 34]. A tyramide signal amplification (TSA) 7-color multiplex fluorescence immunohistochemistry kit (Abcarta, PS017) was used, which contains TSA-fluorophore 425, TSA-fluorophore 488, TSA-fluorophore 532, TSA-fluorophore 594, TSA-fluorophore 633, TSA-fluorophore 680, DAPI, HRP-conjugated goat anti-rabbit and mouse polymer and TSA signal amplification solution (details are provided in Table S1).

Image acquisition and analysis

All stained slides were scanned and imaged using the HEIDSTAR fluorescent scanning system (v23.10.30). Subsequently, the acquired image files were seamlessly opened and fused using the HALO image analysis platform (v3.6.4134.362). The HiphPlex FL module was used for the quantitative analysis. The initial step in image analysis involves cell segmentation and phenotyping. Nuclei were identified and segmented utilizing the DAPI counterstain in conjunction with a set of user-defined parameters, including the nuclear contrast threshold, minimum nuclear intensity, and maximum image brightness. After nuclear segmentation, the parameters for membrane and cytoplasmic detection were refined. Adjustments were made to parameters such as the maximum cytoplasm radius, membrane segmentation aggressiveness to discern individual cells, and cell size range to exclude outliers, ensuring only cells within a specified area (μm^2) were analyzed. After satisfactory optimization of the cell segmentation parameters, cell phenotyping was performed. Phenotype positivity, based on individual dyes or co-expression signals, was adjusted using precise thresholds for positivity and completeness, tailored to each dye. Finally, quantitative analyses were performed using regions of interest.

Statistical analysis

The data were analyzed using SPSS version 26.0 (IBM Corp., Armonk, NY) and R software (version 4.2.1). The differences between two categorical variables were examined

using the Chi-squared or Fisher's exact test. Two or multiple continuous variables were compared using the Student's t-test or one-way analysis of variance, respectively. Non-parametric variables were compared using the Mann-Whitney test or Kruskal-Wallis test. Building a multivariable logistic model is a systematic process. Continuous variables, including AFP and NLR, were assessed for their diagnostic performance in discriminating between patients with and without liver metastasis. Optimal thresholds were determined through Receiver Operating Characteristic (ROC) curve analysis by maximizing Youden's index. Based on these optimal thresholds, the continuous variables were then categorized into categorical variables for further analysis. Categorical candidate variables were screened by univariate analysis using the Chi-square test or Fisher's exact test. Variables with a p value < 0.1 on univariate analysis were considered for multivariable logistic analysis and a nomogram was established. Kaplan-Meier curves were employed to analyze survival data, complemented by the log-rank test for assessing differences between groups. To calculate the hazard ratio (HR) and the 95% confidence interval (CI), univariate and multivariate Cox regression analyses were performed. Statistical significance was set at $P < 0.05$ (two-sided).

References

1. Mei, Y., Li, M., Wen, J., Kong, X. & Li, J. Single-cell characteristics and malignancy regulation of alpha-fetoprotein-producing gastric cancer. *Cancer Med.* **12**, 12018–12033 (2023).
2. Lu, J. *et al.* Whole-exome sequencing of alpha-fetoprotein producing gastric carcinoma reveals genomic profile and therapeutic targets. *Nat. Commun.* **12**, 3946 (2021).
3. Cibulskis, K. *et al.* Sensitive detection of somatic point mutations in impure and heterogeneous cancer samples. *Nat. Biotechnol.* **31**, 213–219 (2013).
4. Saunders, C. T. *et al.* Strelka: accurate somatic small-variant calling from sequenced tumor-normal sample pairs. *Bioinforma. Oxf. Engl.* **28**, 1811–1817 (2012).
5. Talevich, E., Shain, A. H., Botton, T. & Bastian, B. C. CNVkit: Genome-Wide Copy Number Detection and Visualization from Targeted DNA Sequencing. *PLoS Comput. Biol.* **12**, e1004873 (2016).
6. Janjigian, Y. Y. *et al.* Genetic Predictors of Response to Systemic Therapy in Esophagogastric Cancer. *Cancer Discov.* **8**, 49–58 (2018).
7. Mayakonda, A., Lin, D.-C., Assenov, Y., Plass, C. & Koeffler, H. P. Maftools: efficient and comprehensive analysis of somatic variants in cancer. *Genome Res.* **28**, 1747–1756 (2018).
8. Ka, S. *et al.* HLAscan: genotyping of the HLA region using next-generation sequencing data. *BMC Bioinformatics* **18**, 258 (2017).
9. McLaren, W. *et al.* The Ensembl Variant Effect Predictor. *Genome Biol.* **17**, 122 (2016).
10. Reynisson, B., Alvarez, B., Paul, S., Peters, B. & Nielsen, M. NetMHCpan-4.1 and NetMHCIIpan-4.0: improved predictions of MHC antigen presentation by concurrent motif deconvolution and integration of MS MHC eluted ligand data. *Nucleic Acids Res.* **48**, W449–W454 (2020).
11. O'Donnell, T. J., Rubinsteyn, A. & Laserson, U. MHCflurry 2.0: Improved Pan-

Allele Prediction of MHC Class I-Presented Peptides by Incorporating Antigen Processing. *Cell Syst.* **11**, 42-48.e7 (2020).
12. Wu, J. *et al.* DeepHLApan: A Deep Learning Approach for Neoantigen Prediction Considering Both HLA-Peptide Binding and Immunogenicity. *Front. Immunol.* **10**, 2559 (2019).
13. Wu, J. *et al.* TSNAdb v2.0: The Updated Version of Tumor-specific Neoantigen Database. *Genomics Proteomics Bioinformatics* **21**, 259–266 (2023).
14. Rizvi, N. A. *et al.* Cancer immunology. Mutational landscape determines sensitivity to PD-1 blockade in non-small cell lung cancer. *Science* **348**, 124–128 (2015).
15. Miller, C. A. *et al.* SciClone: inferring clonal architecture and tracking the spatial and temporal patterns of tumor evolution. *PLoS Comput. Biol.* **10**, e1003665 (2014).
16. Jiang, J. *et al.* Integrative analysis reveals a clinicogenomic landscape associated with liver metastasis and poor prognosis in hepatoid adenocarcinoma of the stomach. *Int. J. Biol. Sci.* **18**, 5554–5574 (2022).
17. Burrell, R. A. *et al.* Replication stress links structural and numerical cancer chromosomal instability. *Nature* **494**, 492–496 (2013).
18. Bhattacharya, S. *et al.* ImmPort: disseminating data to the public for the future of immunology. *Immunol. Res.* **58**, 234–239 (2014).
19. Li, D., Zhang, X. & Jiang, L. Molecular mechanism and potential therapeutic targets of liver metastasis from gastric cancer. *Front. Oncol.* **12**, 1000807 (2022).
20. Wong, G. Y. M., Diakos, C., Hugh, T. J. & Molloy, M. P. Proteomic Profiling and Biomarker Discovery in Colorectal Liver Metastases. *Int. J. Mol. Sci.* **23**, 6091 (2022).
21. Liao, Y.-L. *et al.* Identification of SOX4 target genes using phylogenetic footprinting-based prediction from expression microarrays suggests that overexpression of SOX4 potentiates metastasis in hepatocellular carcinoma. *Oncogene* **27**, 5578–5589 (2008).
22. Yoshihara, K. *et al.* Inferring tumour purity and stromal and immune cell admixture from expression data. *Nat. Commun.* **4**, 2612 (2013).
23. Barbie, D. A. *et al.* Systematic RNA interference reveals that oncogenic KRAS-driven cancers require TBK1. *Nature* **462**, 108–112 (2009).
24. An, Y. *et al.* Tertiary lymphoid structure patterns aid in identification of tumor microenvironment infiltration and selection of therapeutic agents in bladder cancer. *Front. Immunol.* **13**, 1049884 (2022).
25. Sautès-Fridman, C., Petitprez, F., Calderaro, J. & Fridman, W. H. Tertiary lymphoid structures in the era of cancer immunotherapy. *Nat. Rev. Cancer* **19**, 307–325 (2019).
26. Cabrita, R. *et al.* Tertiary lymphoid structures improve immunotherapy and survival in melanoma. *Nature* **577**, 561–565 (2020).
27. Liberzon, A. *et al.* The Molecular Signatures Database (MSigDB) hallmark gene set collection. *Cell Syst.* **1**, 417–425 (2015).
28. Ayers, M. *et al.* IFN-γ-related mRNA profile predicts clinical response to PD-1 blockade. *J. Clin. Invest.* **127**, 2930–2940 (2017).

29. Jiang, P. *et al.* Signatures of T cell dysfunction and exclusion predict cancer immunotherapy response. *Nat. Med.* **24**, 1550–1558 (2018).
30. Wolf, F. A., Angerer, P. & Theis, F. J. SCANPY: large-scale single-cell gene expression data analysis. *Genome Biol.* **19**, 15 (2018).
31. Becht, E. *et al.* Dimensionality reduction for visualizing single-cell data using UMAP. *Nat. Biotechnol.* (2018) doi:10.1038/nbt.4314.
32. Hu, C. *et al.* CellMarker 2.0: an updated database of manually curated cell markers in human/mouse and web tools based on scRNA-seq data. *Nucleic Acids Res.* **51**, D870–D876 (2023).
33. Jin, S. *et al.* Inference and analysis of cell-cell communication using CellChat. *Nat. Commun.* **12**, 1088 (2021).
34. Hu, C. *et al.* Tertiary Lymphoid Structure-Associated B Cells Enhance CXCL13+CD103+CD8+ Tissue-Resident Memory T-Cell Response to Programmed Cell Death Protein 1 Blockade in Cancer Immunotherapy. *Gastroenterology* **166**, 1069–1084 (2024).

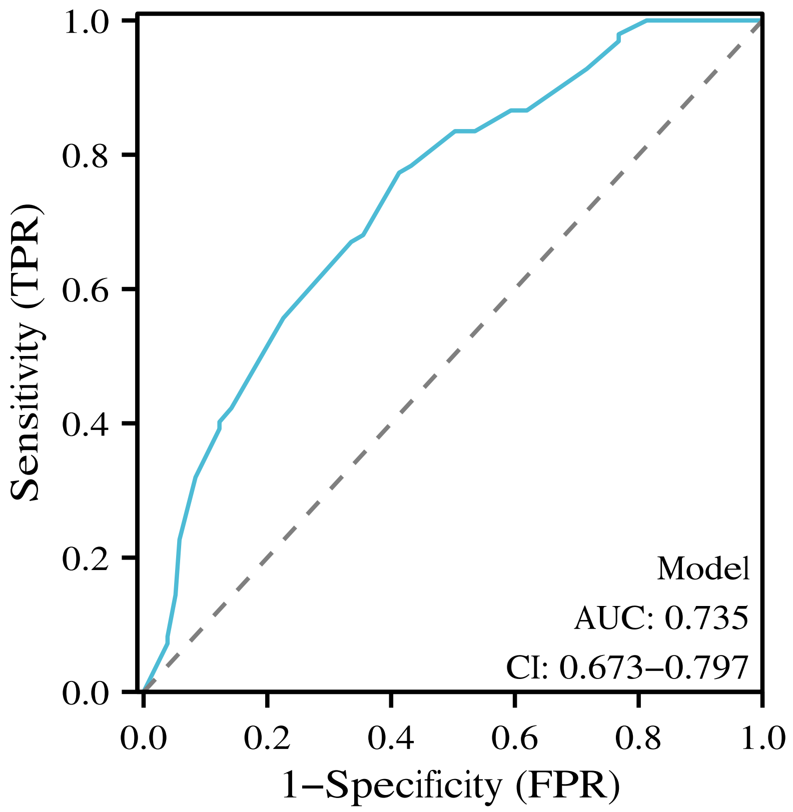

Figure S1. The ROC curves of the nomogram model. AUC, area under the curve; CI, Confidence Interval; TPR, True Positive Rate; FPR, False Positive Rate.

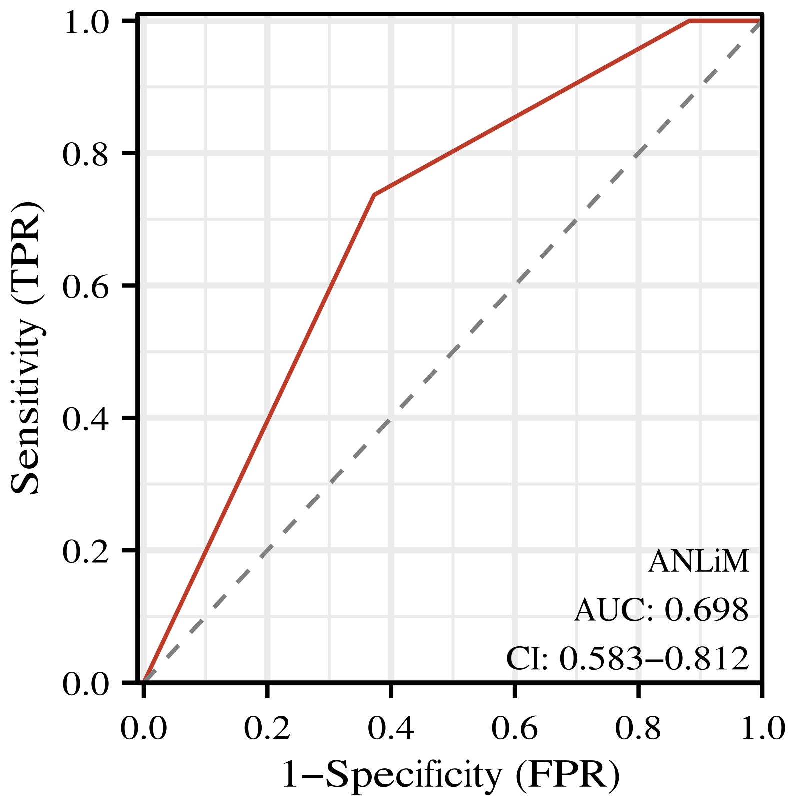

Figure S2. Validation of the predictive value of the ANLiM score for synchronous liver metastasis in the validation cohort. AUC, area under the curve; CI, Confidence Interval; TPR, True Positive Rate; FPR, False Positive Rate.

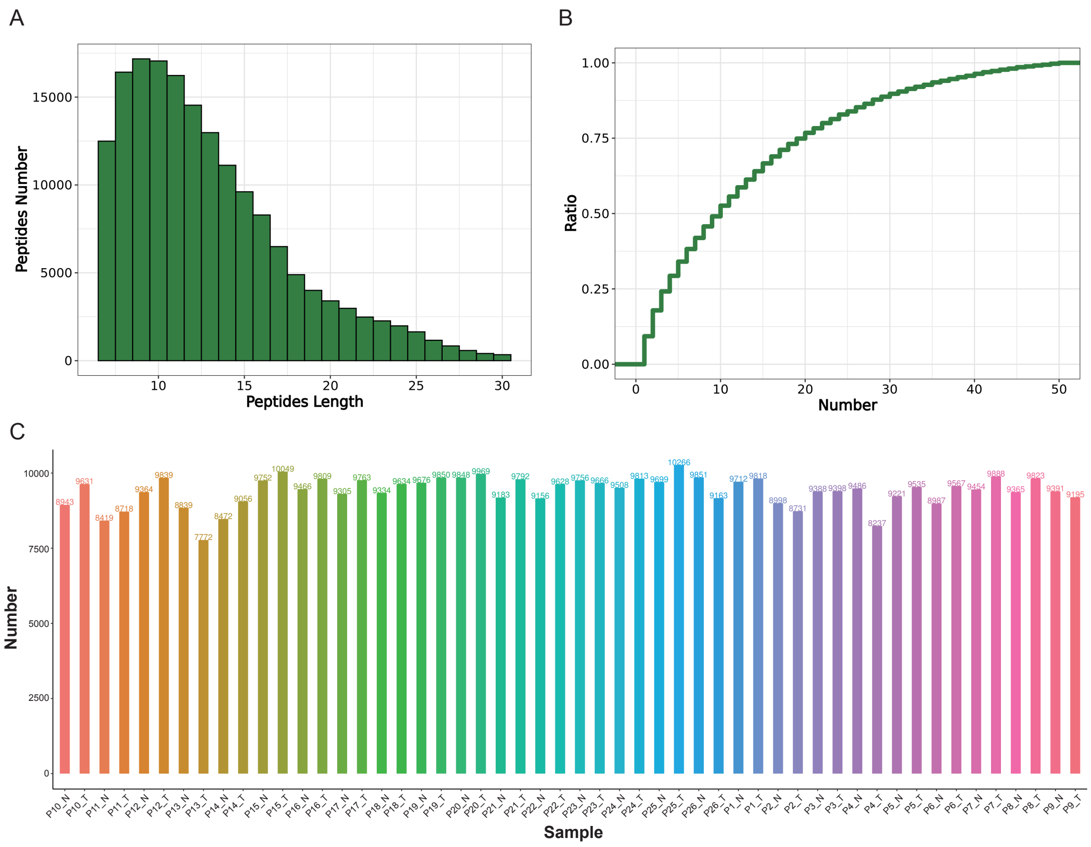

Figure S3. The quality control of proteomic data. (A) Distribution of Peptide Lengths; (B) Cumulative Distribution Curve; (C) Overview of protein identification in samples.

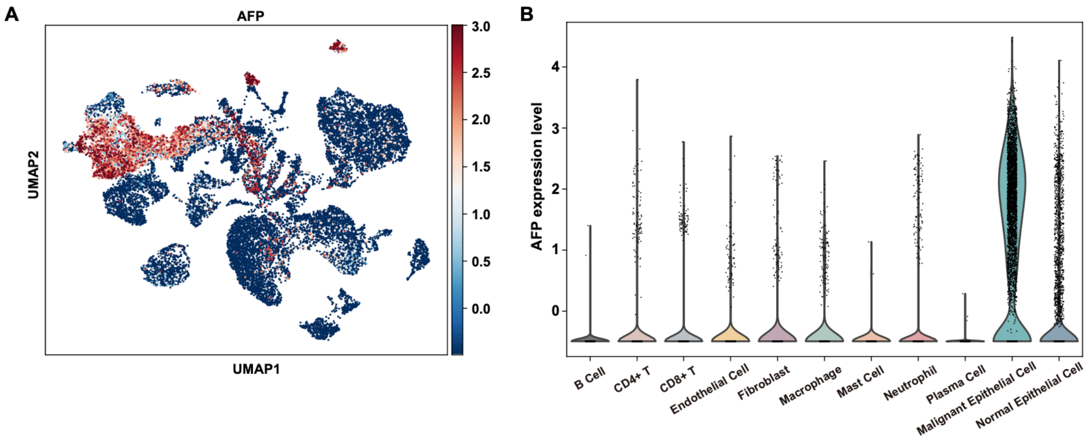

Figure S4. The expression levels of AFP in different cell types. (A) The mRNA expression and distribution of alpha-fetoprotein (AFP) in various cell types; (B) Comparison of AFP expression across different cell types.

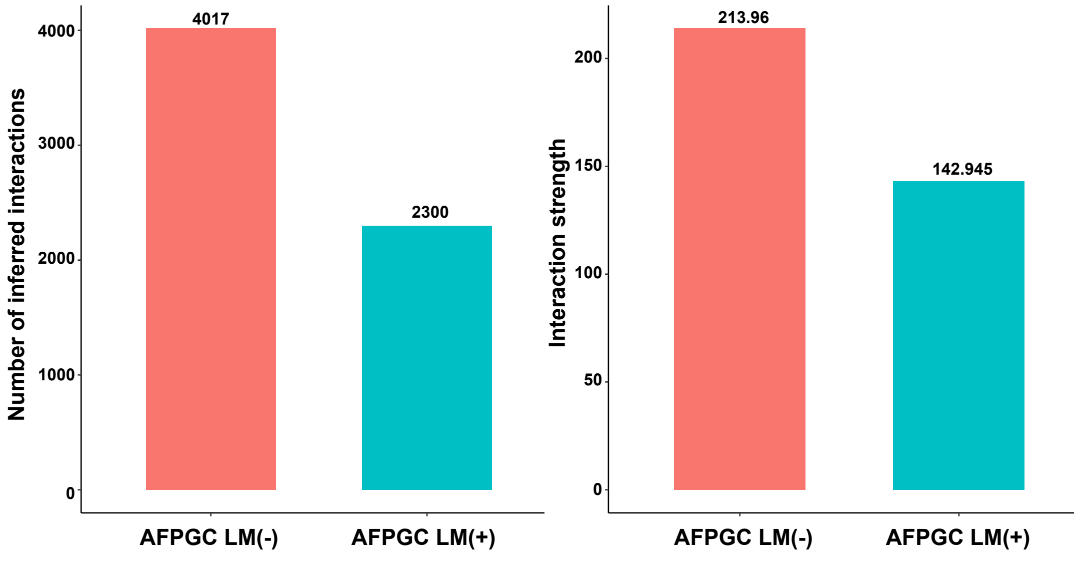

Figure S5. The total number and intensity of interactions between cell clusters were identified using CellChat. LM, liver metastasis; LM(-), the absence of liver metastasis; LM(+), the presence of liver metastasis.

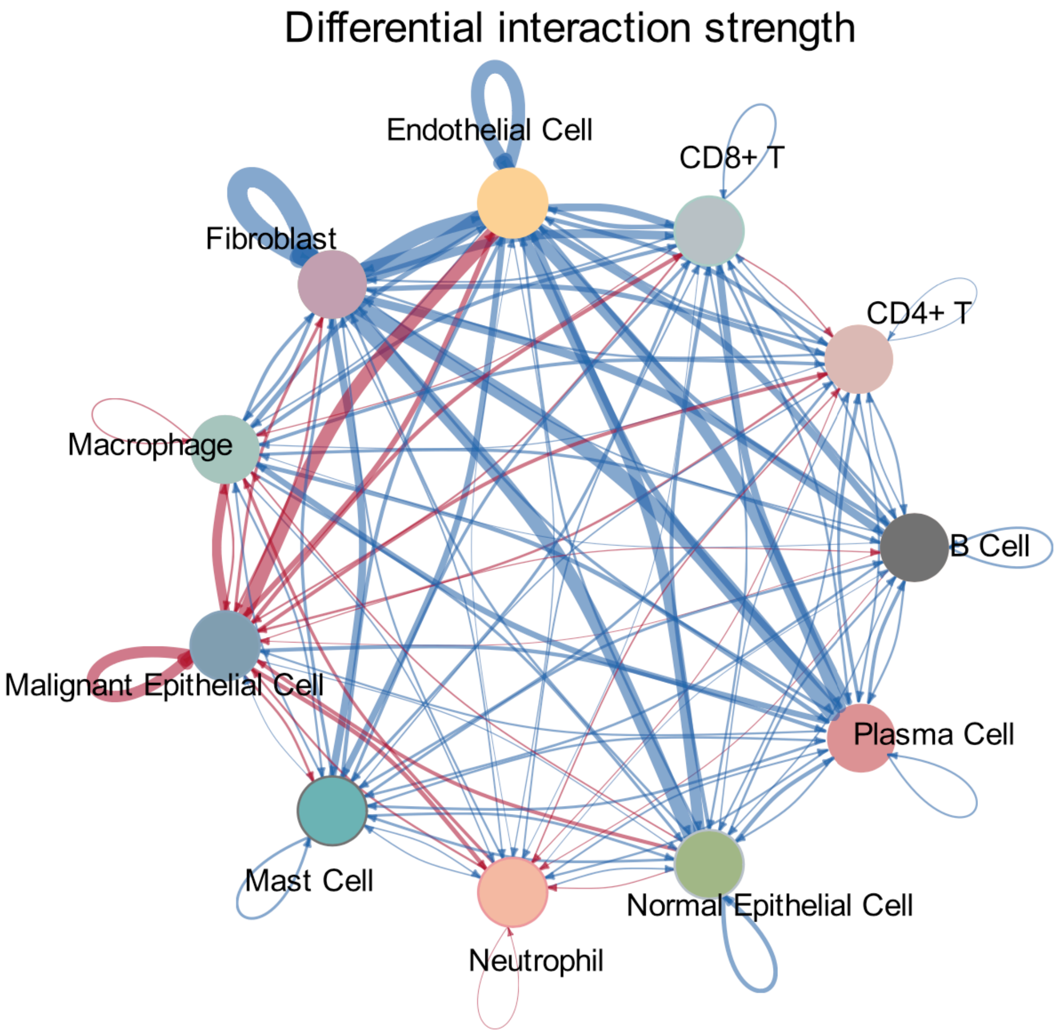

Figure S6. The differences in the intensity of interactions between the AFPGC LM(+) and LM(-) groups. LM, liver metastasis; LM(-), the absence of liver metastasis; LM(+), the presence of liver metastasis. Red (blue) edges indicate that the intensity of interactions increase (decrease) in the LM(+) group.

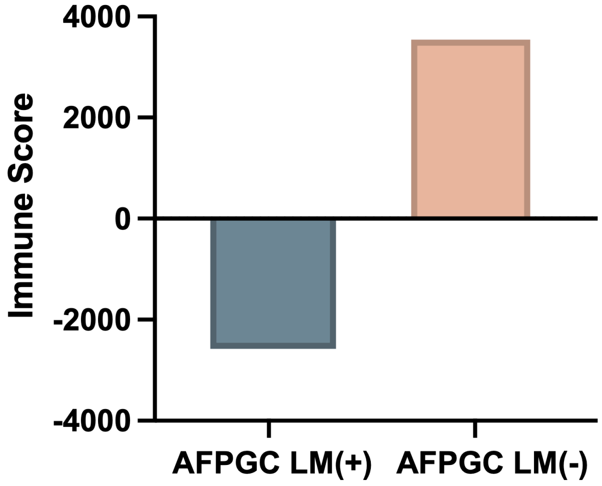

Figure S7. Comparison of ImmuneScores between AFPGC LM(-) and AFPGC LM(+) groups using single-cell transcriptomic data. LM, liver metastasis; LM(-), the absence of liver metastasis; LM(+), the presence of liver metastasis.

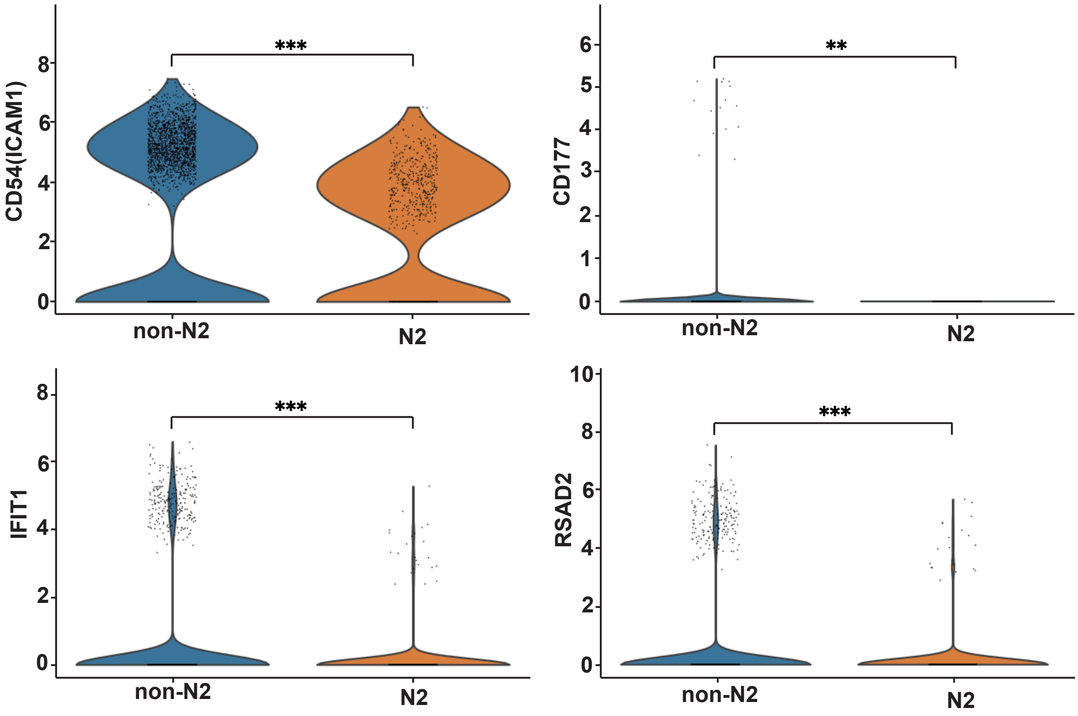

Figure S8. The expression of markers for N1 neutrophils (CD54 and CD177) and markers for interferon-stimulated neutrophils (IFIT1

and RSAD2).

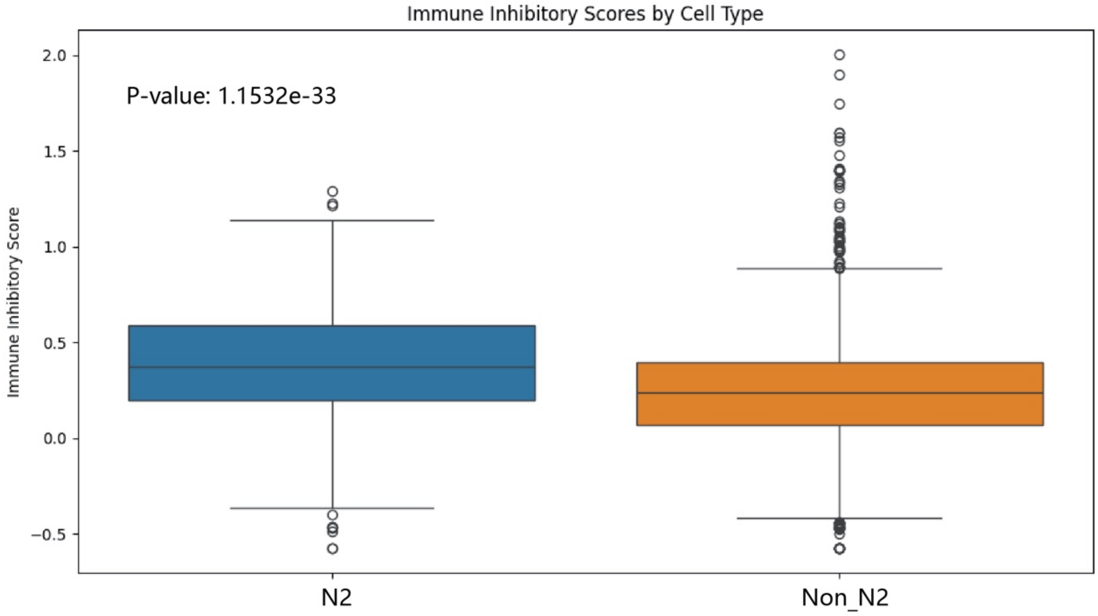

Figure S9. The immune inhibitory scores for the N2 neutrophils and for the non-N2 neutrophils.

Table S1. Markers and reagents for multiplex immunofluorescence (mIF) analysis

Panel	Staining cycle	Marker	Company	Cat. No.	Antibody dilution	Fluorophore	Fluorophore dilution
Panel 1	1	CD66b	Abcam	ab25354	1/20000	TSA 680	1/200
	2	PD-L1	Abcepta	AD80167	Ready-to-use	TSA 633	1/200
	3	CD11c	Abcarta	PA555	Ready-to-use	TSA 594	1/200
	4	MMP9	Abcarta	PA583	Ready-to-use	TSA 532	1/200
	5	TGF-β	Abcarta	PA492	Ready-to-use	TSA 488	1/200
	6	HLA-DR	Abcarta	PA254	Ready-to-use	TSA 425	1/200
Panel 2	1	CD23	Abcarta	PA351	Ready-to-use	TSA 680	1/200
	2	CD21	Abcarta	PA208	Ready-to-use	TSA 633	1/200
	3	CD20	Abcepta	AD80206	Ready-to-use	TSA 594	1/200
	4	CD4	Abcarta	PA285	Ready-to-use	TSA 532	1/200
	5	CD8	Abcarta	PA577	Ready-to-use	TSA 488	1/200
	6	FoxP3	Abcarta	PA448	Ready-to-use	TSA 425	1/200

Table S2. The number of AFPGC patients from each hospital in the external cohort.

Centers	No. of Patients
Lishui Central Hospital	20
The Second Affiliated Hospital, Wenzhou Medical University	15
Jinyun People's Hospital	13
Dongyang People's Hospital	10

Table S3. The comparison of the clinicopathological characteristics between the AFPGC and non-AFPGC cohorts

Characteristics	AFPGC （N=317）	Non-AFPGC （N=777）	*P*
**Age**			<0.001[a]
Median	66	63	
Range	23-88	16-93	
**Gender**			<0.001
Female	69 (21.8%)	255 (32.8%)	
Male	248 (78.2%)	522 (67.2%)	
**TNM**			<0.001
I	19 (6%)	177 (22.8%)	
II/III	145 (45.7%)	427 (54.9%)	
IV	153 (48.3%)	173 (22.3%)	
**Tumor site**			<0.001
Upper	87 (27.6%)	116 (15.3%)	
Middle	58 (18.4%)	184 (24.3%)	
Lower	170 (54%)	457 (60.4%)	
Others	2	20	
**Differentiation**			<0.001
Moderate/Well	36 (12.9%)	176 (23.6%)	
Poor	243 (87.1%)	571 (76.4%)	
NA	38	30	
**Hepatoid differentiation**			<0.001
Yes	87 (31.4%)	3 (0.4%)	
No	190 (68.6%)	744 (99.6%)	
NA	40	30	
**Her2 status**			<0.001
Positive	51 (26.8%)	58 (10.7%)	
Negative	139 (73.2%)	486 (89.3%)	
NA	127	70	
**Liver metastasis**			<0.001
Yes	132 (41.6%)	88 (11.3%)	
No	185 (58.4%)	689 (88.7%)	
**Peritoneal metastasis**			<0.001
Yes	23 (7.3%)	168 (21.6%)	
No	294 (92.7%)	609 (78.4%)	

a, *P* values was from Student's t test and the others were from chi-squared test or Fisher's exact test. *P* was significant at < 0.05.

Table S4. Univariate analysis for factors associated with liver metastasis of AFPGC patients in the multicenter cohort

Characteristics	Liver metastasis (-) (N=205)	Liver metastais (+) (N=112)	*P*
**Age**			0.943
< 60	52 (25.4%)	28 (25%)	
≥ 60	153 (74.6%)	84 (75%)	
**Gender**			0.68
Female	47 (22.9%)	22 (19.6%)	
Male	158 (77.1%)	90 (80.4%)	
**Serum AFP level**			<0.001
≤ 136.40ng/ml	118 (57.6%)	35 (31.2%)	
> 136.40ng/ml	87 (42.4%)	77 (68.8%)	
**Serum CEA level**			0.063
< 5.00ng/ml	95 (47.5%)	41 (36.6%)	
≥ 5.00ng/ml	105 (52.5%)	71 (63.4%)	
NA	5	0	
**Serum CA199 level**			0.029
< 37.00U/ml	142 (72.4%)	67 (60.4%)	
≥ 37.00U/ml	54 (27.6%)	44 (39.6%)	
NA	9	1	
**Serum CA125 level**			<0.001
< 35U/ml	132 (83.5%)	64 (62.7%)	
≥ 35U/ml	26 (16.5%)	38 (37.3%)	
NA	47	7	
**NLR**			<0.001
≤ 2.14	86 (44.1%)	18 (17%)	
> 2.14	109 (55.9%)	88 (83%)	
NA	10	6	
**Tumor site**			0.766
Upper	51 (24.9%)	29 (25.9%)	
Middle	38 (18.5%)	20 (17.9%)	
Lower	114 (55.6%)	63 (56.2%)	
Others	2 (1%)	0 (0%)	

The optimal cutoff values of AFP and NLR were decided according to the receiver operating characteristic (ROC) curve. The upper normal values (5 ng/mL, 37 U/mL and 35U/mL) were used as the cutoff values for CEA , CA199 and CA125.

Table S5. A comparison of basic clinicopathological characteristics between the multicenter cohort and the validation cohort.

Characteristics	Multicenter cohort (N = 317)	Validation cohort (N = 70)	*P*
**Age**			0.136
Median	66	68	
Range	23-88	35-82	
**Gender**			0.14
Female	69 (21.8%)	21 (30%)	
Male	248 (78.2%)	49 (70%)	
**TNM**			0.153
I	19 (6%)	0 (0%)	
II	34 (10.7%)	8 (11.4%)	
III	111 (35%)	30 (42.9%)	
IV	153 (48.3%)	32 (45.7%)	
**Serum AFP level**			0.092
Median	158.5	246.2	
Range	20.20-92900.00	22.7-164000	
**Serum CEA level**			0.902
Median	7.1	6.25	
Range	0.50-22748.80	0.50-4537.2	
**Serum CA199 level**			0.259
Median	13.2	16.6	
Range	0.5-305800	2.00-194145.00	
**Serum CA125 level**			0.544
Median	16.1	15.8	
Range	2.60-3661.00	4.4-372.6	
**NLR**			0.27
Median	2.57	2.94	
Range	0.78-17.50	0.95-10.21	
**Tumor site**			0.96
Upper	87 (27.6%)	18 (26.5%)	
Middle	58 (18.4%)	12 (17.6%)	
Lower	170 (54%)	38 (55.9%)	
Others	2	0	
**Differentiation**			0.831
Poor	243 (87.1%)	59 (88.1%)	
Moderate/Well	36 (12.9%)	8 (11.9%)	
NA	38	3	
**Hepatoid differentiation**			0.516
Yes	87 (31.4%)	16 (27.1%)	
No	190 (68.6%)	43 (72.9%)	
NA	40	11	

NA, not avaialble.

Table S6. The Comparison of gene alteration frequencies among the three groups.

Gene	AFPGC LM(-)		AFPGC LM(+)		MSKCC-GC LM(+)		AFPGC LM(-) vs AFPGC LM(+)	MSKCC-GC LM(+) vs AFPGC LM(+)
	No. of MUT	No. of WT	No. of MUT	No. of WT	No. of MUT	No. of WT	*P* value	
ERBB2	10	28	9	11	40	49	0.15	0.996
ERBB3	4	34	2	18	12	77	> 0.999#	> 0.999#
CCNE1	7	31	10	10	20	69	0.012	0.013
CCND1	6	32	3	17	3	86	> 0.999#	0.074#
ATM	2	36	0	20	9	80	0.54#	0.206#
TP53	28	10	16	4	65	24	0.751#	0.777#
MYC	8	30	5	15	15	74	0.732	0.395
PIK3CA	1	37	3	17	14	75	0.114#	> 0.999#
PTEN	3	35	0	20	10	79	0.544#	0.203#
SMAD4	0	38	0	20	26	63	> 0.999#	0.003#
ARID1A	6	32	1	19	15	74	0.403#	0.295#
ARID1B	2	36	1	19	6	83	> 0.999#	> 0.999#
CSMD3	10	28	5	15	15	74	0.913	0.395

# P values were from two-sided Fisher's exact test and the others were from chi-square test, and were significant at < 0.05. MUT, the presence of gene alterations, including somatic mutations or copy number variations; WT, the absence of gene alterations, including somatic mutations and copy number variations; LM, liver metastasis; LM(-), the absence of liver metastasis; LM(+), the presence of liver metastasis; MSKCC, Memorial Sloan Kettering Cancer Center.

Table S7. The differentially expressed proteins between LM(+) and LM(-) in AFPGC

Gene	logFC	adj.P.Val
ADH1A	1.67330977	0.001790593
HMGA2	1.61781074	0.001465719
GATM	1.56896603	0.00484206
KBTBD6	1.56110604	0.000195954
ICE1	1.46943464	0.005993368
SCML2	1.45343238	0.002196102
ALDOC	1.44605358	0.002650133
NPM1	1.34258664	0.00179536
AATF	1.32897476	0.001295521
SDHAF2	1.32603315	0.002637624
PDF	1.32326729	0.006527928
DDO	1.32325211	0.010358909
TKTL1	1.32103028	0.005145922
LLPH	1.31229636	0.00557358
RPS6KA6	1.29282944	0.009790571
PDZK1	1.2901351	0.015254337
PRORP	1.27933748	0.002543549
HSPD1	1.27578908	0.0099657
KCTD1	1.26304725	0.010144837
FAT4	1.25710293	0.002887816
ITGAE	1.25631826	0.015435378
MOCS3	1.24827802	0.018478515
PMPCA	1.24654349	0.001562932
KCTD15	1.23291874	0.011114674
HSD17B2	1.22676102	0.006989677
EFNA1	1.22517593	0.008918409
PNMA3	1.21759815	0.023604723
DNAJC2	1.21680804	0.007575418
LCLAT1	1.21560235	0.014724778
IGF2BP2	1.20705195	0.026280345
NOP2	1.20390907	0.010229683
MLF2	1.20039237	0.01622696
YARS2	1.18544064	0.023669198
NIPSNAP1	1.18122419	0.021964648
UCHL1	1.18046459	0.036645333
CT45A5	1.17806447	0.026782374
PHC1	1.17282004	0.005704536
DDX54	1.17001604	0.022171789
HSPE1	1.16945766	0.011148445
UBFD1	1.16494945	0.023186213
UBAC2	1.16049389	0.015542392
C2orf72	1.15691805	0.029570291

EAPP	1.15520985	0.009892942
SURF6	1.15409802	0.013120094
SOWAHA	1.15263469	0.026161657
PLPP6	1.14780871	0.01311897
DHCR7	1.14510988	0.033341754
RRS1	1.14507578	0.014432024
SUGCT	1.14381782	0.020824772
TNN	1.14209433	0.031536909
CCDC154	1.12413639	0.029120748
TUBB1	1.12334284	0.038900552
SERPINA5	1.12052994	0.037473398
RBM19	1.11524922	0.012141874
ITGA2B	1.10807124	0.047580129
EBNA1BP2	1.10707143	0.016526043
AS3MT	1.10554556	0.044376132
GEMIN8	1.10418544	0.004951961
ANPEP	1.10143252	0.036144787
PMPCB	1.09156189	0.007210736
SPON2	1.09119258	0.026037507
ZBTB16	1.08475125	0.011935778
MCAT	1.08297998	0.021959274
METTL3	1.08268626	0.030174833
HMGCS2	1.08109577	0.036320139
USP11	1.07345547	0.043060897
MBNL3	1.06690971	0.04979503
CEP131	1.05977697	0.025323499
LYRM4	1.05620527	0.012250359
PTPRF	1.05364032	0.037170679
EPHX1	1.05136627	0.048340993
REXO4	1.04763723	0.019559319
DDX52	1.04631581	0.027964746
BRIX1	1.04573794	0.015673074
FAF2	1.04099362	0.029868581
PID1	1.04040969	0.02888512
EHHADH	1.03801127	0.044710007
KLHL20	1.03220135	0.031840441
ADH6	1.02647289	0.031124327
NOP53	1.02120935	0.030426724
STAU1	1.01746791	0.031341788
IL1RAP	1.01329814	0.040828983
NOL7	1.01101201	0.038429953
RSL1D1	1.00987501	0.026673812
LUZP1	-1.000118	0.046494008
SGPL1	-1.0008228	0.047878591
AIF1	-1.0011747	0.046744998

FGL2	-1.0014253	0.036590631
PLAA	-1.0015106	0.049314214
AGTRAP	-1.0020681	0.045471413
MORC3	-1.0035161	0.013586789
UGT8	-1.0036	0.047839724
CARS1	-1.0044798	0.002803452
GZMH	-1.0048384	0.030253685
GART	-1.0055146	0.047218491
ENPEP	-1.0060635	0.042065542
TRAF3IP3	-1.0067743	0.008646601
SEPTIN4	-1.0088022	0.025186872
MAN1A2	-1.0090618	0.011485034
INTS5	-1.009499	0.011805251
GRAMD1A	-1.009524	0.024707186
MFSD1	-1.0096957	0.042673948
PIK3CD	-1.0103525	0.034032962
CYGB	-1.0113858	0.030993982
SPIN1	-1.0118762	0.039536618
ASAP2	-1.0119653	0.01015828
CPSF7	-1.0121048	0.041528676
MMRN2	-1.012917	0.01035743
IGKV3D-11	-1.013218	0.022689761
MORN1	-1.0134126	0.007192662
PSMA1	-1.0135559	0.011324909
PSMA4	-1.0147069	0.018108832
HDAC4	-1.0148187	0.002593596
GNLY	-1.0149866	0.031276642
PSMA2	-1.0157853	0.022363491
POLR2G	-1.0161152	0.016489814
FNBP1	-1.0174909	0.016716126
RASIP1	-1.0175903	0.008053533
CBL	-1.0179022	0.049412347
EVL	-1.0185438	0.024184922
CDH5	-1.0191879	0.010739891
MCC	-1.0198433	0.0144612
VSIR	-1.0208631	0.012101567
CPNE3	-1.02106	0.036127142
ARHGDIB	-1.0217371	0.049903211
PARP9	-1.0217508	0.023030489
TNPO1	-1.0225132	0.030092899
RNPEP	-1.0229593	0.018104893
PIK3CA	-1.0237953	0.036055428
AKT1	-1.0238219	0.036609704
SPG21	-1.0239827	0.037162944
CST7	-1.0239898	0.012638918

APOL6	-1.0241203	0.018457035
GLRX	-1.0242808	0.01071634
SIGLEC1	-1.0248131	0.040497611
DOCK10	-1.0257268	0.013374694
STK38	-1.0259714	0.034038033
GNAI2	-1.0269824	0.01585873
CAP1	-1.0274883	0.035285435
OSBPL5	-1.0279384	0.011192073
FYB1	-1.0287182	0.030739625
TTC39C	-1.0287705	0.047755024
SAMHD1	-1.0291551	0.03018841
RHOG	-1.0300826	0.010855139
PKN1	-1.0309501	0.02055678
PARP10	-1.0322776	0.022205356
AP2A2	-1.0325591	0.017039827
TMED8	-1.0358933	0.011017128
PLEKHO2	-1.0374619	0.039572343
SERPINB9	-1.0377164	0.015119194
ATP13A3	-1.0386437	0.036771384
CBLB	-1.0390606	0.012760014
TBC1D23	-1.0394056	0.014437432
ABRACL	-1.0397843	0.019561096
OSBPL3	-1.0400201	0.036630572
SUN2	-1.0401262	0.040743961
TBC1D10B	-1.0408163	0.032887816
LOC102723407	-1.0411662	0.021399601
STAG2	-1.0417283	0.029450972
NECAP2	-1.0418723	0.025993165
VPS41	-1.0425177	0.038974953
DYNLT1	-1.0448546	0.030957948
LSM2	-1.0455033	0.021024624
GYS1	-1.0460505	0.044124985
PIK3R1	-1.0465965	0.035775363
PSMA5	-1.046692	0.008820559
MCOLN1	-1.0468256	0.018114254
IGKV1-17	-1.0469013	0.019890326
LRRC32	-1.0477337	0.013044386
EOGT	-1.0478162	0.039252616
MX1	-1.0479081	0.039178148
AGAP3	-1.0482225	0.008838114
IGKV4-1	-1.048374	0.006460752
UBLCP1	-1.0499544	0.025238933
BORCS8	-1.0502085	0.003933327
ITGB2	-1.0508249	0.044542641
SEMA4A	-1.0512291	0.002349553

RBM5	-1.051497	0.012646911
TRIM22	-1.05258	0.021223754
TSPAN14	-1.0528262	0.037458253
IGHV3-49	-1.0530419	0.006035642
TBC1D10C	-1.0534378	0.011960177
PTK2B	-1.0540017	0.010352217
RAVER1	-1.0555545	0.037753045
ARHGAP15	-1.0556616	0.014546935
GNG5	-1.0561229	0.020457164
IGKV2D-29	-1.0569752	0.022578607
DHPS	-1.0579249	0.027576798
SASH3	-1.0584928	0.007226444
IKBKE	-1.058877	0.007049471
NUDT3	-1.0591798	0.043475337
STX6	-1.0604845	0.032833197
EDNRA	-1.0609568	0.031205984
WDR61	-1.0618425	0.003907262
MTA2	-1.0620365	0.03194719
VCP	-1.0621443	0.015111546
FYN	-1.0621595	0.009746605
NUP188	-1.0624445	0.017831671
ARFGEF1	-1.0631722	0.032763037
VPS28	-1.0653383	0.01332178
RABEP1	-1.066126	0.015716167
DAGLB	-1.0672254	0.03631159
DOCK2	-1.0675503	0.009393135
KCNAB1	-1.0675934	0.037348763
PFDN1	-1.0681852	0.03998897
RNF213	-1.0700161	0.024206646
PTPN6	-1.0702368	0.008802767
CYTH4	-1.0702659	0.007673992
CNPY3	-1.0710313	0.015117076
SERPINB8	-1.0711035	0.037163597
SELENOS	-1.0716128	0.036810695
LZIC	-1.0717476	0.037376562
SPAG9	-1.0722522	0.038791618
HAT1	-1.0734823	0.021073249
PSMB8	-1.0739893	0.008558313
LYN	-1.0745731	0.019221936
NUP205	-1.0750983	0.022823255
PPP2R5B	-1.0769568	0.027478966
IGHG1	-1.0772143	0.011846926
ZFYVE1	-1.0778579	0.010308817
MAPK13	-1.0779877	0.036961581
ATG2A	-1.078178	0.025259832

CTSS	-1.078194	0.048937247
DAPK2	-1.0782768	0.016366535
CASP2	-1.0789442	0.003045629
TOR3A	-1.0791743	0.015904025
ELOA	-1.0794678	0.009461439
DOCK9	-1.0803769	0.029351194
TBC1D22B	-1.0804212	0.012148233
SPPL2A	-1.0817604	0.049000362
CLEC2B	-1.0817934	0.014104615
FAM50A	-1.0821928	0.026552267
ICAM3	-1.0825328	0.031170604
LSM4	-1.0825411	0.02865685
SBF1	-1.0826941	0.009392588
UBE2G1	-1.0836053	0.006575159
MX2	-1.0837569	0.023413744
UBE2E2	-1.0845322	0.031730509
PSMD6	-1.0845927	0.027577177
DEF6	-1.0854467	0.015090548
SIPA1	-1.0866843	0.0289208
ULK3	-1.0867407	0.002490718
WDFY3	-1.0873254	0.017286037
ANKRD13A	-1.0879163	0.012327534
ADA2	-1.0895455	0.016084012
PARP14	-1.0898931	0.016880419
GK	-1.0899937	0.040190708
TNPO3	-1.0902122	0.033344329
ARHGAP4	-1.0903877	0.022747164
TRPV2	-1.0915869	0.026081742
VAV1	-1.0926476	0.018970779
IGHV3-30	-1.0946605	0.03407665
SLC43A2	-1.0947773	0.017723612
VPS11	-1.0956007	0.007491816
HLA-DMA	-1.0959921	0.022687736
TSG101	-1.0961953	0.017594526
HLA-C	-1.0963563	0.019090092
CASP3	-1.09665	0.004400439
HLA-E	-1.0966637	0.009167112
PAAF1	-1.0974282	0.007812478
KPNB1	-1.0975728	0.02038242
CLTA	-1.098355	0.018236943
PKNOX1	-1.1002458	0.00411918
INTS15	-1.1005772	0.002166748
RPA3	-1.1007867	0.016067433
SH3BP1	-1.1014184	0.004031365
CYLD	-1.1016455	0.01360868

RASSF5	-1.1019117	0.018818722
MYO1F	-1.1028428	0.035534213
NCKAP1L	-1.1033842	0.015424673
ETS1	-1.1034783	0.006134538
UBE2B	-1.1037592	0.013083518
CHUK	-1.1038517	0.007812305
BAZ1B	-1.103935	0.022995826
MED16	-1.104673	0.010128817
IGHV3-15	-1.1056292	0.037612343
GRK6	-1.1079847	0.00627968
UACA	-1.108156	0.016481941
KIDINS220	-1.1090708	0.013781352
LAMTOR2	-1.1097961	0.006515958
HCK	-1.1101695	0.030929965
ITPRIP	-1.1107129	0.020965946
SYNE1	-1.1108847	0.030559542
STXBP2	-1.1121684	0.015332032
TTC7A	-1.1123292	0.00960203
PPP4R1	-1.1128216	0.025193448
USP48	-1.1131082	0.029288674
PSMA7	-1.1132576	0.019867373
SPRYD3	-1.1149843	0.010159109
BLVRA	-1.1153872	0.023533938
ELMO1	-1.1155663	0.005461031
MIS12	-1.1169327	0.017395094
DNAJC8	-1.1171553	0.008795231
PTPN7	-1.1172731	0.006167922
ABHD16A	-1.118092	0.013098093
STK11IP	-1.1185997	0.012392764
IGLV1-51	-1.119925	0.013683672
ASH2L	-1.1199318	0.019343276
ALOX15	-1.1210285	0.010802565
MSR1	-1.1215539	0.018211119
DYSF	-1.1222582	0.038578306
TDP1	-1.1254496	0.003908543
AP1S2	-1.1266061	0.022881247
APOBEC3G	-1.1268748	0.02724986
NUMA1	-1.128513	0.036882925
KCTD21	-1.1286631	0.020051619
SNAP23	-1.1291132	0.018029613
PLXNC1	-1.1295504	0.02201176
GLMN	-1.1300829	0.028298292
WIPI1	-1.1301108	0.014830761
TMEM259	-1.1320029	0.002214817
ARHGEF1	-1.1324301	0.018207867

ACAP1	-1.1340639	0.010104655
AGTPBP1	-1.134318	0.013259816
ERLEC1	-1.1348083	0.020532892
GSDMD	-1.1360551	0.006338782
GIMAP4	-1.1362338	0.012120535
ESRRA	-1.1369398	0.006491132
CASP8	-1.1372378	0.020530856
PLCB2	-1.1373808	0.023762097
VPS35L	-1.1413624	0.007316094
KANK3	-1.1421044	0.000587205
TAPBP	-1.1432542	0.027307169
PODXL	-1.1435245	0.021348143
BRK1	-1.1439939	0.007428083
PITPNA	-1.1445385	0.006169805
ARMC6	-1.1452492	0.021570693
TCAF1	-1.1461576	0.02698903
IKBKG	-1.1470168	0.015623458
IGKV6D-21	-1.1476003	0.023400375
PPP3CB	-1.149214	0.01152577
FAS	-1.1494059	0.008556412
HGSNAT	-1.1509113	0.020103579
DRAP1	-1.1525197	0.008869074
LCP2	-1.1526548	0.015404384
IGHV3-7	-1.1529108	0.007810289
ZMIZ1	-1.1534443	0.003672432
ABCF2-H2BK1	-1.1543105	0.018894113
FMNL1	-1.1549547	0.021052715
FES	-1.1552583	0.022473647
STRIP1	-1.1555604	0.009686339
OSBPL10	-1.1557727	0.022323965
PDCL3	-1.1562623	0.008715085
PI4K2A	-1.1565406	0.021566122
GBP4	-1.1565785	0.020710476
CSK	-1.1568156	0.002989035
MAPK14	-1.1569746	0.018244736
IKBKB	-1.1572392	0.01017444
SP140	-1.1584416	0.000975252
STING1	-1.1588279	0.015038283
CUTC	-1.1599616	0.001875707
PRF1	-1.1630565	0.005600611
ALPL	-1.1641961	0.01049282
DOK3	-1.1651375	0.019270538
GIMAP5	-1.166776	0.012782774
LCK	-1.1669389	0.018860783
DYRK1A	-1.1669802	0.016345739

SP100	-1.1678733	0.02383677
DTX3L	-1.1686787	0.012278766
IGSF6	-1.1693491	0.013555279
ELOC	-1.1736576	0.021531862
DENND1C	-1.1759467	0.001654773
TRABD	-1.176274	0.006764587
GIMAP1	-1.1770079	0.0105368
NRF1	-1.1777446	0.009803991
CYBC1	-1.1813074	0.019444759
CD300LF	-1.1827596	0.007340896
ARPC4	-1.1838847	0.010106908
TEX264	-1.1862658	0.015900958
PIK3C2A	-1.186279	0.016926561
MLH1	-1.1863898	0.008856571
MSN	-1.1885293	0.015557778
ARHGAP25	-1.1904485	0.014912626
MCM3AP	-1.1905631	0.007675156
BMP2K	-1.1921866	0.006464047
TMEM62	-1.193204	0.024375273
ANXA6	-1.1942015	0.017828605
WAS	-1.1945403	0.009612922
CC2D1B	-1.1947923	0.002846645
LYST	-1.1953655	0.002089746
ARPC3	-1.1955253	0.00836137
TRIM21	-1.196446	0.008562807
CYBB	-1.1964842	0.019674062
KIAA0930	-1.1995628	0.005374503
ACTR2	-1.2013695	0.008665815
RASA2	-1.2017879	0.002994378
ADGRE5	-1.2022901	0.011853378
IL18	-1.2043976	0.014864204
STX7	-1.2056488	0.019169736
ASAP1	-1.2103506	0.020396233
RASAL3	-1.2137072	0.004602264
IFI30	-1.2153917	0.019665818
RABIF	-1.2170995	0.012648984
B2M	-1.21739	0.003052386
GIT1	-1.2175616	0.009260572
LSM3	-1.2181197	0.009899484
SPAG7	-1.2187677	0.009979094
CYRIB	-1.2188848	0.009012246
OSTM1	-1.219025	0.010607966
TAP2	-1.2191657	0.020143927
CD101	-1.2204948	0.008887987
STK17B	-1.2206482	0.001297566

TRAPPC4	-1.2207796	0.002491011
CCZ1B	-1.223889	0.004220296
ARPC5	-1.2239319	0.021478013
MTHFD2	-1.2249796	0.014025779
RBBP7	-1.2265168	0.009017113
ARPC2	-1.2265977	0.008414984
CASP4	-1.2283497	0.008334118
KIF5B	-1.2289013	0.021396205
APOBEC3C	-1.2290728	0.006863858
DTX2	-1.2299255	0.006529231
DOCK8	-1.2302303	0.007418533
SDS	-1.2304993	0.01220752
RRAGA	-1.2306511	0.011010912
EIF4E2	-1.2315748	0.008198438
CD2	-1.235438	0.009703567
INPP5K	-1.2385375	0.001912483
XRN1	-1.2394984	0.012552869
SLC29A3	-1.2395604	0.016789574
MED18	-1.2405168	0.008123013
LGMN	-1.2411343	0.008060297
CD53	-1.2414078	0.00281759
WARS1	-1.2416795	0.019461944
ELOB	-1.2423878	0.013484255
RBBP4	-1.249216	0.006342348
ZHX3	-1.2492417	0.007712343
INTS3	-1.2513286	0.009943458
DOK2	-1.2520539	0.013112315
EIF2B1	-1.2530641	0.01402405
TCIRG1	-1.253276	0.014788363
ARHGAP30	-1.2570526	0.009405782
IGKV1-12	-1.2588378	0.015648666
SIRPA	-1.2612918	0.009253805
TXLNA	-1.2615235	0.012608269
LBR	-1.2622573	0.006658309
ARHGEF6	-1.2627947	0.005076919
LRCH1	-1.263743	0.001881886
LMF2	-1.2693972	0.011144732
VPS39	-1.2711218	0.013346186
GMIP	-1.2713474	0.01117507
ATP6V1A	-1.2723204	0.014684098
SWAP70	-1.2751671	0.00238147
KPNA1	-1.2761763	0.009749379
TASOR	-1.2768335	0.005317978
SMCHD1	-1.279393	0.004189796
ARHGAP45	-1.2807056	0.006045429

TRAF1	-1.2807915	0.002139244
PPP1R9B	-1.2809643	0.009729512
PPP2R5E	-1.2830821	0.011801542
SEH1L	-1.2843817	0.003603539
NOS3	-1.2856971	0.003471561
CORO1A	-1.2893168	0.003022409
STX3	-1.2894403	0.017883636
MICAL1	-1.2909184	0.008294272
DCK	-1.2930726	0.001323493
GZMB	-1.2952613	0.00347387
CLIP1	-1.2956038	0.014849548
LCP1	-1.2986888	0.007695533
GIMAP8	-1.3094931	0.001401792
TRAM2	-1.3126475	0.001740489
MTHFR	-1.320932	0.008956061
ITGAL	-1.3230825	0.005342284
LSM1	-1.3258108	0.001122898
UBE2Q1	-1.3266807	0.004280272
LAP3	-1.3286592	0.009844348
GUCY1A2	-1.3395375	0.00313251
MPHOSPH8	-1.3446718	0.004226896
PTPRC	-1.34787	0.002770576
CYTH1	-1.3575775	0.006152561
SNX14	-1.3700449	0.000552523
MEF2D	-1.3716758	0.001296865
TAP1	-1.3731085	0.006716151
WDR45	-1.373945	0.001681548
UBE2D1	-1.3746013	0.002224139
MYO9B	-1.374602	0.007059548
DCAF7	-1.3762634	0.003998791
ITGA4	-1.3783682	0.003015907
SLFN5	-1.3790641	0.001583469
PTPRE	-1.383902	0.007180203
DNASE2	-1.3851501	0.003779803
MAPKAPK3	-1.3888397	0.003766147
HLA-A	-1.3912628	0.002805722
RIPK1	-1.3912982	0.001151529
ADPGK	-1.3924004	0.006241787
GRK2	-1.4012764	0.002446335
SLFN11	-1.4174163	0.001941128
PLEKHM2	-1.4237497	0.001722674
RGS19	-1.4260051	0.000942385
VAV3	-1.4276485	0.001392269
ATP6V0D1	-1.4312119	0.007770854
NAGK	-1.435208	0.004889859

ARRB2	-1.4371076	0.006821894
SYNJ1	-1.4435106	0.002869142
RRAGC	-1.4456529	0.001615564
CDC40	-1.4559225	0.002323476
SPATA13	-1.4626214	0.001096506
CD93	-1.4640896	0.001562358
PGD	-1.4688888	0.005213964
DNAJC13	-1.4700872	0.005316748
OSBPL8	-1.4701652	0.005321736
ADPRH	-1.4796078	0.001613648
TRIM26	-1.4796344	0.002241781
SELENON	-1.4933809	0.000904772
PECAM1	-1.4973327	0.000869033
UBE2L6	-1.501908	0.001385526
PLCL2	-1.5055725	0.001201789
TRAPPC3	-1.5073037	0.000128854
ICMT	-1.5122983	0.002429388
LITAF	-1.5233076	0.003841148
HVCN1	-1.5476257	0.001063313
CALCRL	-1.5714345	0.000150327
SLC17A5	-1.5855494	0.002617041
ADGRE2	-1.6011873	0.000205221
NADK	-1.6335405	0.000173987
MAPKAPK2	-1.6893775	0.000335046
TXNL4A	-1.7152775	0.000584092
TMX3	-1.7415852	0.000779732

FC, fold change; LM, liver metastasis; LM(-), the absence of liver metastasis; LM(+), the presence of liver metastasis.

Table S8. Clinicopathologic characteristics of AFPGC patients in this study and cell numbers after quality control.

Group	Patient ID	Age	Gender	Serum AFP (ng/mL)	Differentiation	Hepatoid differentiation	Lauren's classification	Cell numbers after quality control
LM(+)	P1	69	Male	1250.8	Moderate/Well	Yes	Intestinal	10027
	P2	76	Male	1108.4	Poor	No	Intestinal	
LM(-)	P3	25	Female	83.9	Poor	NA*	Diffuse	11373
	P4	68	Male	188.9	Moderate/Well	NA	Mixed	

LM, liver metastasis; LM(-), the absence of liver metastasis; LM(+), the presence of liver metastasis; *NA, not available.

Table S9. The composition and proportion distribution of immune cells in AFPGC.

Immune cell type	AFPGC	Proportion	AFPGC LM(+)	AFPGC LM(-)
**Neutrophil**	3991	0.405	771	3220
**CD4+ T**	2079	0.211	958	1121
**CD8+ T**	1951	0.198	875	1076
**Macrophage**	783	0.079	487	296
**Plasma Cell**	626	0.064	4	622
**B Cell**	316	0.032	22	294
**Mast Cell**	108	0.011	34	74
**Sum**	9854	1	3151	6703

Table S10. The comparison of clinicopathologic variables between the two groups.

Characteristics	Liver metastasis (-) (N=19)	Liver metastais (+) (N=30)
**Age**		
< 60	8 (42.1%)	8 (26.7%)
≥ 60	11 (57.9%)	22 (73.3%)
**Gender**		
Female	5 (26.3%)	6 (20.0%)
Male	14 (73.7%)	24 (80.0%)
**Differentiation**		
Moderate/Well	3 (17.6%)	3 (11.1%)
Poor	14 (82.4%)	24 (88.9%)
NA	2	3
**Tumor site**		
Lower	12 (63.2%)	19 (63.3%)
Middle	2 (10.5%)	4 (13.3%)
Upper	5 (26.3%)	7 (23.3%)
**dMMR/MSI-H**		
dMMR	2 (11.8%)	0 (0.0%)
pMMR	15 (88.2%)	20 (100.0%)
NA	2	10
**ANLiM**		
0	4 (21.1%)	0 (0.0%)
1	6 (31.6%)	9 (30.0%)
2	9 (47.4%)	21 (70.0%)
**TNM stage**		
III	19 (100%)	0 (0.0%)
IV	0 (0.0%)	30 (100%)
**Chemoimmunotherapy regimens**		
XELOX/FOLFOX/SOX + PD-1	14 (73.7%)	26 (86.7%)
FLOT + PD-1	3 (15.8%)	0 (0%)
AS + PD-1	2 (10.5%)	4 (13.3%)

XELOX/FOLFOX/SOX represent oxaliplatin combined with fluorouracil-based drugs, specifically: oxaliplatin combined with capecitabine, oxaliplatin combined with 5-FU, and oxaliplatin combined with Tegafur; FLOT, chemotherapy with 5-fluorouracil, oxaliplatin, and docetaxel; AS, paclitaxel plus S-1; PD-1, anti-PD-1 drugs, such as Nivolumab, Pembrolizumab and Sintilimab et al. NA, not avaialble.
